# Pharmacological assessment of the extract and a novel compound of *Bacillus velezensis* DM derived from the rhizosphere of *Datura metel* L. with microbial molecular screening

**DOI:** 10.1186/s12906-025-04879-x

**Published:** 2025-04-26

**Authors:** Mohamed A. Awad, Shahenda Mahgoub, Hesham S. M. Soliman, Sherif F. Hammad

**Affiliations:** 1 Biotechnology Program, Institute of Basic and Applied Science, Egypt-Japan University of Science and Technology (E-JUST), New Borg El-Arab City, Alexandria, 21934 Egypt; 2 Botany and Microbiology Department, Faculty of Science, Sohag University, Sohag, 82524 Egypt; 3 Department of Biochemistry and Molecular Biology, Faculty of Pharmacy, Helwan University, Ain- Helwan, Cairo, 11795 Egypt; 4 Department of Pharmacognosy, Faculty of Pharmacy, Helwan University, Ain-Helwan, Cairo, 11795 Egypt; 5 Department of Pharmaceutical Chemistry, Faculty of Pharmacy, Helwan University, Ain-Helwan, Cairo, 11795 Egypt; 6 PharmD Program, Egypt-Japan University of Science and Technology (E-JUST), New Borg El-Arab City, Alexandria, 21934 Egypt

**Keywords:** *Datura metel* L., Rhizosphere bacteria, *Bacillus velezensis* DM, Biosynthetic genes, *In vitro* pharmacology

## Abstract

**Background:**

Rhizosphere bacteria were considered a prospective reservoir of bioactive compounds with significant pharmacological efficacy.

**Methods:**

From the rhizosphere of *Datura metel* L., *Bacillus velezensis* DM was isolated and characterized using 16 S rRNA. PCR screening and sequencing were conducted to identify genes related to bioactive metabolite production. The extraction of secondary metabolites from the bacterial strain was performed via a fermentation process. The ethyl acetate extract of the propagated strain was subjected to fractionation and purification through various chromatographic techniques. The characterization of the isolated compounds was accomplished using different spectroscopic methods, such as 1D and 2D-NMR. An MTT test was conducted to assess the cytotoxic activity of bacterial extract on MCF-7, HepG-2, and HCT-116 cells. Furthermore, its pure compound (**1**) was tested for its cytotoxicity on HCT-116 and a normal cell (THLE2) to test its safety for normal cells. Apoptosis was identified through flow cytometry on HCT-116 cells after double-staining with PI and annexin V-FITC. The antioxidant action of bacterial extract was assessed through DPPH and ABTS assays. Furthermore, anti-inflammatory evaluations were carried out employing lipoxygenase (5-LOX) and cyclooxygenase (COX-2) inhibition.

**Results:**

The NCBI GenBank database has effectively incorporated the 16 S rRNA gene sequence of *Bacillus velezensis* DM under the accession number OR364492. Polyketide synthase and two lipopeptide genes for surfactin and iturin A were effectively detected by PCR, and their sequences were included in the Genbank database. A novel compound, 5,6-di(methylamino)hex-5-ene-1,2,3-triol (**1**), was successfully separated from the strain. Bacterial extract demonstrated significant cytotoxic activity against the evaluated cancer cells, exhibiting the most pronounced effect on HCT-116 cells. Compound (**1**) showed promising cytotoxic potential against HCT-116 cells with a higher selectivity index (2.5) towards cancer cells in comparison to Doxorubicin (1.49). Apoptosis assay showed that bacterial extract caused apoptosis about 14 folds compared to the control HCT-116 cells. Furthermore, it showed a potent anti-inflammatory outcome (IC_50_ = 1.927 µg/mL) and antioxidant activity at IC_50_ of 76.8 µg/mL.

**Conclusion:**

This study revealed the possible pharmacological effects of secondary metabolites generated by *Bacillus velezensis* DM, making it a valuable resource for isolating bioactive compounds with potential therapeutic and biomedical uses.

**Supplementary Information:**

The online version contains supplementary material available at 10.1186/s12906-025-04879-x.

## Background

The rhizosphere is a dynamic ecosystem where microorganisms fiercely compete for resources necessary for survival [1]. This diverse group of microorganisms is essential in enhancing plant development, aiding nutrient uptake, and maintaining plant health [2]. Consequently, an increasing focus has been on discovering environmentally friendly substitutes for harmful chemicals and pesticides [3].

The *Datura* genus is a member of the Solanaceae family and is distributed across several global locations. There are 14 plant species within this genus, but the most used for medicinal purposes are *D. metel* L., *D. innoxia*, and *D. stramonium*. Despite its toxicity, *D. metel* L. has been traditionally utilized to treat a variety of ailments, including epilepsy, cardiac disease, diarrhea, and even madness [4]. This perennial herb thrives in warmer climates and is often grown in tropical and subtropical areas because of its striking flowers [5]. *D. metel* L. has numerous medicinal properties, such as astringent, bitter, germicidal, acrid, anti-phlogistic, anodyne, narcotic, antiseptic, and soothing. Its various parts can treat skin diseases, painful menstruation, hemorrhoids, wounds, burns, and skin ulcers. Furthermore, the herb has beneficial effects such as being a narcotic, anti-tussive, antispasmodic, bronchodilator, and anti-asthmatic, making it a versatile natural remedy for many ailments [6]. *Datura metel* L. contains a variety of phytochemicals, such as withanolides, daturaolone, datumetine, daturglycosides, ophiobolin A, baimantuoluoline A, and others [4]. Fascinatingly, as the plant evolves, its seeds accumulate a higher concentration of numerous substances, including alkaloids, tannins, cardiac glycosides, flavonoids, carbohydrates, amino acids, and phenolic substances [7, 8].

The *Datura* rhizosphere contains a diverse microbiome with beneficial and potentially novel bacterial strains. The bioactive compounds in *Datura* exudates promote the growth of specific microbes that exhibit plant growth-promoting (PGP) properties, antimicrobial activity, and biodegradation potential. These traits make them valuable for agriculture and bioremediation, as some microbes may help control soilborne pathogens by producing antibiotics or competing for nutrients. For example, *Sphingomonas sanguinis* DM was previously isolated from the rhizosphere of *Datura metel* [9]. Several bacterial strains identified in the *Datura* root microbiome include *Bacillus*, *Chitinophaga*, *Pseudomonas*, *Streptomyces*, *Rhizobium*, and *Flavobacterium* [10].

*Bacillus velezensis*, a Gram-positive, aerobic, and endospore-forming bacterium, is incredibly beneficial and can be found in various environments, including the rhizosphere of plants. Strains of *Bacillus velezensis* have been identified as pivotal in promoting plant development. After colonizing its root, these strains can induce a distinct physiological state known as the primed state in the host plant. Priming the plant makes it better prepared to respond to biotic or abiotic stress more efficiently [11]. A previous study found that *Bacillus velezensis* could be a biocontrol agent to protect plants from various pathogens [12]. However, recent findings have revealed that it also acts as an antagonist against fish and aquatic pathogens [13]. With its antibacterial, antioxidant, anti-inflammatory, antidiabetic, and anticancer properties, *B. velezensis* could be a promising therapeutic agent [14]. This bacterium possesses a significant genetic potential for producing cyclic lipopeptides like surfactin, bacillomycin-D, fengycin, and bacillibactin, as well as polyketides such as macrolactin, bacillaene, and difficidin. It also produces beneficial metabolites like siderophore, bacteriocins, plantazolicin, dipeptide bacilysin, and other volatile organic compounds such as acetoin and 2,3-butanediol [15].

Microorganisms have beneficial properties, such as producing secondary metabolites that can positively impact the environment [16]. The genetic makeup of microbes and their specific symbiosis influence these properties significantly [17]. It was found that most natural products belong to polyketides, made by polyketide synthases (PKSs) [18]. For instance, discodermolide is typically synthesized using bacterial type I modular polyketide synthase [19]. Lipopeptides are antimicrobial compounds synthesized by bacteria, including *Bacillus* strains [20]. They have a cyclic structure, a low molecular weight, and consist of a hydrophilic head with 7–10 amino acids and a hydrophobic fatty acid tail. *Bacillus* strains release three main lipopeptide groups: surfactin, iturin, and fengycin families [21]. Iturins and fengycins have potent antifungal properties, whereas surfactins have strong antibacterial properties [22]. Studying microorganisms’ host specificity is vital to identifying unique secondary metabolites with potential ecological or therapeutic benefits. Finding a suitable bioactive strain depends on the existence of a gene responsible for secondary metabolites. Combining bioactivity and gene screening is effective [23].

Based on the challenges mentioned above, this study aimed to highlight the pharmacological role of *Bacillus velezensis* DM derived from the rhizosphere of *Datura metel* L. through various applications, including evaluating antioxidant and anti-inflammatory activities. Furthermore, the cytotoxicity of the bacterial extract was assessed using the MTT test, followed by the induction of apoptosis test. Additionally, the study attempted to provide molecular evidence of *Bacillus velezensis* DM by investigating its host specificity to detect PKS and lipopeptide genes responsible for synthesizing bioactive metabolites.

## Materials and methods

### Plant material [9]

*Datura metel* was collected from the new Borg Elarab city, Alexandria, Egypt, in December 2021. The identification of the plant material was carried out by Dr. M. Gibali, Lecturer of plant taxonomy, NRC, Cairo, Egypt, and a specimen of the plant code (Pharm-EJUST 2021-05) was kept in the Pharmacognosy department, PharmD program, Egypt-Japan University of Science and Technology. No permission was required for plant sampling from public land around the University Campus.

### Materials

Dichloromethane, methanol, *n*-hexane, and ethyl acetate were obtained from El Nasr Pharmaceutical Chemicals Co, Egypt. The necessary supplies, including trypan blue dye, MTT, and dimethyl sulfoxide (DMSO), were acquired from Sigma in St. Louis, MO, USA. Also, L-glutamine, gentamycin, the fetal bovine serum, RPMI-1640, HEPES buffer solution, and 0.25% Trypsin-EDTA were procured from Lonza in Belgium.

### Isolation of bacteria from the rhizosphere of *Datura metel* L

To gather rhizosphere samples, it was necessary to carefully uproot the root system of *Datura metel* L. and store it in a cool container for transport to the laboratory. Upon arrival, one gram of soil was ground using a sterile pestle and mortar, and serial dilutions (10X) were prepared. 0.1 mL of each dilution, ranging from 10^− 3^ to 10^− 5^, was then evenly distributed on tryptone soy agar plates to isolate the rhizosphere bacteria [24]. Following an overnight incubation period at 37 °C, well-defined colonies that had formed on the growth medium were carefully chosen for additional refinement. These selected colonies were then re-streaked on fresh plates containing tryptone soy agar medium to guarantee their purity and obtain individual cell colonies. Ultimately, the bacterial cultures were allowed to grow for 24 h at 37 °C and preserved in 20% glycerol at -80 °C [25].

### Isolation of bacterial DNA

A micro-centrifuge tube with a volume of 1.5 mL was utilized to transfer the enriched bacterial culture. One milliliter of the culture was added to the tube, and the cell suspension was centrifuged for 10 min at 14,000 × g. After centrifugation, the supernatant was discarded carefully. The resulting pellet was resuspended in 300 µL of DNase-RNase-free distilled water (Sigma, Sigma–Aldrich s.r.l., Gallarate, Milan, Italy) by vortexing. The tube underwent another round of centrifugation for 5 min at 14,000 × g. Upon removal of the supernatant, the remaining pellet was blended with 200 µL of DNase-RNase-free distilled water (Sigma) by vortexing. The suspension was subsequently heated at 100 °C for 15 min and swiftly cooled on ice. The mixture was centrifuged once more for 5 min at 14,000 × g at 4 °C. The supernatant was carefully transferred to a new microcentrifuge tube, incubated again for 10 min at 100 °C, and chilled immediately on ice. A DNA template for PCR was obtained by extracting five microliters of the supernatant [26].

### Bacterial characterization by 16 S rRNA sequencing and phylogenetic analysis

With the assistance of SolGent Company, based in Daejeon, South Korea, sequencing of the 16 S rRNA gene from the bacterial isolate was conducted. SolGent utilized its specialized purification beads to purify the extracted DNA. The polymerase chain reaction (PCR) method was employed to amplify the ribosomal rRNA gene (rDNA) using two universal bacterial primers, 27 F and 1492R, in the reaction mix. Electrophoresis was utilized with a nucleotide marker (100 base pairs) on a 1% agarose gel to ascertain the size of the purified PCR products (amplicons). After extracting the resulting bands, dideoxynucleotides (ddNTPs) were integrated into the reaction mixture. This process enabled the sequencing of the bands in both the sense and antisense directions using the 27 F and 1492R primers [27].

### Phylogenetic analysis

The BLAST tool at the NCBI was utilized to determine the similarity between the isolate’s DNA sequence and those available in the GenBank database, followed by the use of the ClustalX program (version 1.81) and the neighbor-joining (NJ) method for aligning the sequences to create a phylogenetic tree or dendrogram [28].

### PCR detection of genes related to bioactive metabolites biosynthesis

The process of amplifying DNA samples in polymerase chain reactions (PCRs) is commonly accomplished using the Taq polymerase protein and a T1 Thermal Cycler (Biometra, Germany). The resulting amplified products were then analyzed using 1% agarose gel electrophoresis. To investigate the molecular properties involved in PKS production in *Bacillus velezensis* DM, degenerate oligonucleotide primers were used to detect the KS domain of modular PKS genes. The following steps and cycles were involved in the reaction: 3 min at 94 °C, 35 cycles at 94 °C for 1 min, 59.1 °C for 1 min, and 72 °C for 1 min, followed by 7 min at 72 °C [23]. Furthermore, the strain underwent a thorough examination to detect the presence of two lipopeptide genes, including surfactin and iturin A. PCR was employed to amplify the lipopeptide gene, starting with an initial denaturation for 5 min at 95 °C, followed by 30 cycles of denaturation (1 min at 94 °C), annealing (1 min at 52 °C for *sfp* gene and 59.7 °C for *ItuD* gene), extension (1 min at 72 °C), and a final extension at 72 °C for 10 min [22]. A detailed list of specific gene primers utilized in PCR screening is provided in Table 1.

**Table 1:** PCR primers for amplification of PKS and lipopeptide genes

Target gene	Primers	Sequences (5’- 3’)	Amplicon size (bp)
KS domain	KS-F KS-R	GCGATGGATCCNCAGCAGCG GTGCCGGTNCCGTGNGYYTC	680
*sfp*	sfp-F sfp-R	ATGAAGATTTACGGAATTTA TTATAAAAGCTCTTCGTACG	675
*ItuD*	ItuD1-F ItuD1-R	GATGCGATCTCCTTGGATGT ATCGTCATGTGCTGCTTGAG	647

### DNA sequencing

The PCR product was purified from a 1% agarose gel using a GeneDireX gel extraction kit (Taiwan). Following this, sequencing was performed by the Macrogen facility in Korea using the Big TriDye sequencing kit (ABI Applied Biosystems) with the KS-F, sfp-F, and ItuD1F forward primers [23].

### Sequence analysis and Genbank accession numbers

Using BioEdit version 5.0.7, the PKS, sfp, and ItuD nucleotide sequences were translated into peptide sequences. The BLAST tool, developed in collaboration with the National Center for Biotechnology Information (NIH, MD, USA), was used to determine the similarity percentage between nucleotide sequences and other sequences. To create a phylogenetic tree, nucleotide sequences were aligned with various sequences obtained from GenBank using Clustal X [29]. The MEGA version 3.1 was employed to construct neighbor-joining phylogenies [30]. All nucleotide sequences obtained from *Bacillus velezensis* DM during this investigation have been submitted to the NCBI GenBank database.

### Solid-state fermentation, working up and isolation of secondary metabolites

Bacterial colonies were first cultivated on a fresh LB plate to prepare the seed culture. These colonies were then carefully transferred to a 250 mL flask containing 50 mL of liquid LB medium and incubated at 37 °C with agitation at 170 rpm for 24 h [31]. Ten 1 L Erlenmeyer flasks were inoculated aseptically with 5 mL of the seed culture for solid-state fermentation. Each flask was filled with a modified rice medium consisting of 100 g of commercial rice and 150 mL of distilled water containing 0.4% yeast extract. After that, the bacterial cultures were incubated under static conditions at 37 °C for 14 days. Following this, the cells were subjected to sonication for 30 min, resulting in the breakdown of the bacterial cells. The propagated media, with a total volume of 1.5 L, was then harvested and extracted through maceration using methanol (1.5 L). Subsequently, the methanol extract was filtered under a vacuum. The resulting aqueous methanol filtrate was then concentrated *in vacuo* using rotary evaporation (Heidolph, Germany) at 40 °C until all the methanol had evaporated. Then, the residual material was suspended in water and subjected to extraction with ethyl acetate until exhaustion. Eventually, the ethyl acetate extract was evaporated till dryness (24.39 gm) and kept for further analysis [32].

### Purification and identification of bacterial metabolites

The ethyl acetate fraction underwent column chromatography with normal phase silica gel (70–230 mesh, Merck, Germany) in a 60 × 3 cm column. The column was eluted using a gradient system of methylene chloride (DCM) and methanol (MeOH), and the eluted fractions were closely monitored on TLC (DC-Alufolien, silica gel 60 F254 matrix, Merck, Germany). The common fractions were collected and dried using a rotatory evaporator under a vacuum below 40⁰C. The solvent strength DCM and MeOH (9.5:0.5) yielded a crude fraction which was subjected to PTLC using a developing system composed of DCM: MeOH (9:1). The isolated compound **1** was detected under a 254 nm UV light, scratched from the plate and extracted by a mixture of methanol and methylene chloride. The solvent was distilled to give the pure compound (9 mg). 1D and 2D-NMR spectra were recorded using the Bruker Avance DRX instrument from Rheinstetten, Germany, which operated at 400 MHz (^1^H NMR) and 100.40 MHz (^13^C NMR). The solvent used for compound **1** was deuterated chloroform (CDCl_3_). To carry out the ESI-MS positive ion acquisition mode, the XEVO TQD triple quadruple instrument (Waters Corporation, Milford, MA01757 USA, mass spectrometer) was used. The ACQUITY UPLC-BEH C_18_ 1.7 μm − 2.1 × 50 mm column was utilized with a solvent system of (A) water containing 0.1% formic acid and (B) acetonitrile, with a flow rate of 0.2 mL/min [33]. Furthermore, Dragendorff reagent is employed to detect the alkaloidal nature of the investigated product.

### Cytotoxic evaluation of *B. velezensis* DM extract on human cancer cell lines

Diverse human cancer cell lines: liver cancer cells (HepG2), colon cancer cell line (HCT-116), and breast cancer cell line (MCF-7) were brought from VACSERA Tissue Culture Unit (Giza, Egypt). RPMI-1640 media enhanced with 10% fetal bovine serum and 50 mg /mL gentamycin were employed to culture the cells. The cells were incubated at 37 °C with 5% CO_2_. To determine the cytotoxic activity, cells were treated with various doses (0–100 µg/ mL) of *B. velezensis* DM extract or Doxorubicin, and their viability compared to untreated controls was assessed [34]. Compound (**1**) cytotoxic potential was evaluated against HCT-116 as well as normal epithelial cells (THLE2) to calculate the selectivity index (SI) as formerly depicted [35].

### Annexin‑V/propidium iodide apoptosis assay

HCT116 cells were treated with IC_50_ concentration of *B. velezensis* DM extract before incubating for 48 h. Then, cells were harvested by trypsinization using trypsin-EDTA solution (200 µL), incubated for 4 min at 37 °C, and washed twice with PBS. Afterward, cells were stained with Annexin V-FITC (5 µL) and PI (5 µL) for 10 min at RT in the dark. Cells were analyzed using a FACS Calibur flow cytometer (BD Biosciences, San Jose, CA) by means of CELLQUEST software (Becton Dickinson Immunocytometry Systems, San Jose, CA) directly after the addition of PI [36].

### Antioxidant assessment of *B. velezensis* DM extract

Antioxidant DPPH assay [34]: To inspect the antioxidant potential of *B. velezensis* DM extract, a methodology reported by Nahar et al. was used compared to Trolox [37].

Antioxidant ABTS assay: the capacity of *B. velezensis* DM extract to scavenge ABTS radical cations was assessed compared to Quercetin at a range of concentrations, including 300, 150, 75, 25, and 10 µg/ mL [38].

### Anti-inflammatory assessment

In vitro 5-lipoxygenase (LOX) inhibitory assay: The ability of *B. velezensis* DM extract to inhibit the 5-LOX was examined compared to the reference drug Zileuton [39].

In vitro cyclooxygenase-2 (COX-2) inhibitory assay: The inhibitory effect of *B. velezensis* DM extract on COX-2 enzyme was evaluated by comparing its ability to that of the reference drug Celecoxib [40, 41].

### Statistical analysis

IC_50_ was calculated by the Graph Pad Prism (version 8, San Diego, CA, USA) (*n* = 3 replicates). Data are presented as mean ± SD.

## Results

### Isolation and molecular characterization of bioactive rhizosphere bacterium

This study aimed to examine the growth of bacteria derived from the rhizosphere of *Datura metel* L. (Solanaceae) on tryptone soy agar plates after 12–24 h. It showed that 35 distinct colonies were obtained and selected for further purification. Bacterial isolates were cultivated, and pure cultures were kept at -80 °C in 20% glycerol. One bacterial isolate was subsequently selected for molecular analysis.

Following morphological analysis, the cells were classified as obligate aerobes or facultative anaerobes, with Gram-positive, cream-white, motile, endospore-forming, and rod-shaped characteristics. Using 16 S rRNA gene sequencing, a potent bacterial strain was identified. The bacterium’s DNA was isolated, and the 16 S rDNA sequence was amplified utilizing universal primers (27 F and 1492R). The ensuing PCR product was meticulously purified and sequenced. Upon acquiring the 16 S rDNA sequence of the selected bacterium, it was cross-referenced with the comprehensive non-redundant BLAST database to identify the most closely related sequences. The BLAST algorithm analysis indicated that the bacterium’s 16 S rDNA sequence exhibited a remarkable similarity percentage of 97.50% with multiple *B. velezensis* sequences present on the BLAST database, with a rationally high score and e-value of zero, as demonstrated in the Additional file.

**Fig. 1:**
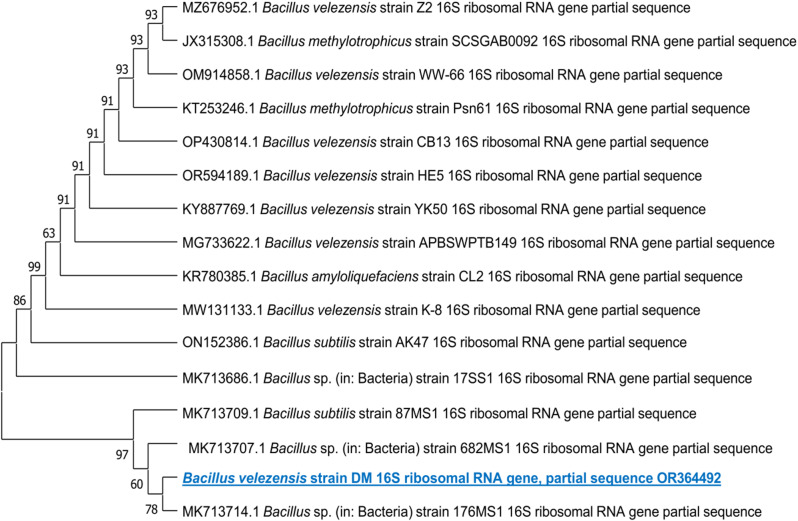
The Phylogenetic tree (1000 bootstrap replicates) depicts the relation between the 16 S rDNA sequence of the rhizosphere *B. velezensis* DM and its possible homologous sequences accessed from the GeneBank

The ClustalX alignment program was utilized to establish a phylogenetic relationship that is readily accessible. The program employed the neighbor-joining algorithm to connect the query sequence with other homologous sequences derived from the BLAST database. A connection must be determined to identify the various species in the gene bank database closely linked to the selected bacterium. The evolutionary connection is presented as a dendrogram (Fig. 1), illustrating a unique rooted evolution. Additionally, the 16 S rRNA gene sequence of *B. velezensis* DM is available in the NCBI GenBank database with the accession number OR364492.

### PCR screening of genes responsible for the synthesis of bioactive metabolites

**Fig. 2:**
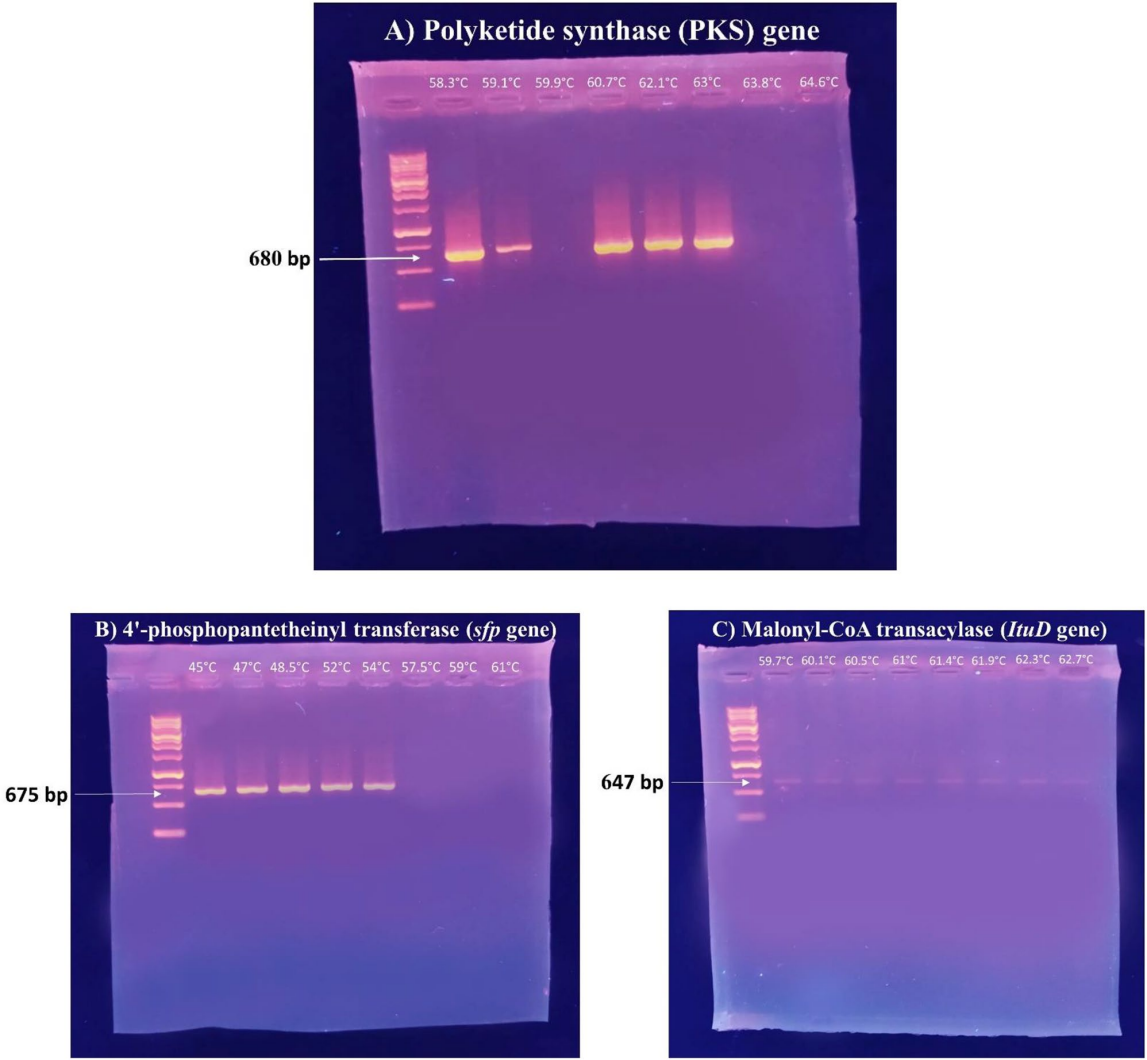
PCR amplification detects the presence of some genes related to the biosynthesis of bioactive metabolites in the rhizosphere strain, *Bacillus velezensis* DM, on a 1% agarose gel electrophoresis using a gradient of eight annealing temperatures to verify the initial PCR results as follows: Polyketide synthase which encodes KS domain (**A**) and lipopeptide genes for surfactin and iturin A, including 4’-phosphopantetheinyl transferase (**B**) and malonyl-CoA transacylase (**C**), respectively

**Fig. 3:**
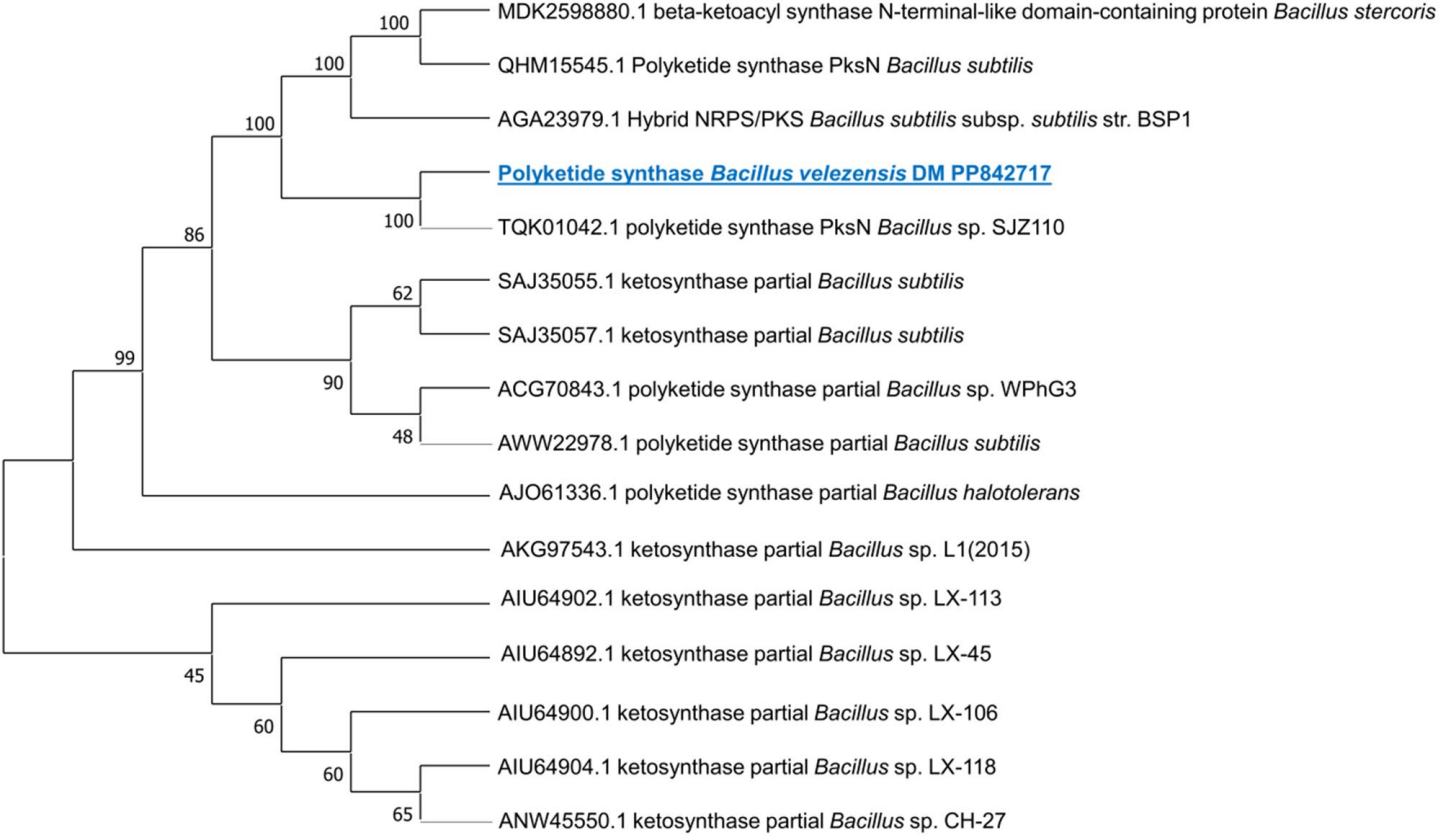
Phylogenetic analysis (1000 bootstrap replicates) of PKS KS domain from *B. velezensis* DM compared to other microorganisms’ diverse KS fragments of *Bacillus subtilis*

**Fig. 4:**
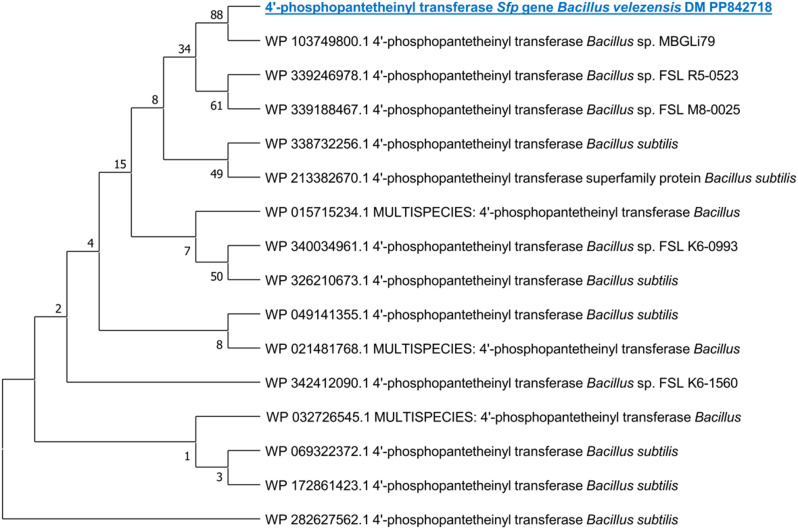
Phylogenetic analysis (1000 bootstrap replicates) of lipopeptide *sfp* gene from *B. velezensis* DM compared with various lipopeptide *sfp* fragments of *Bacillus* sp

**Fig. 5:**
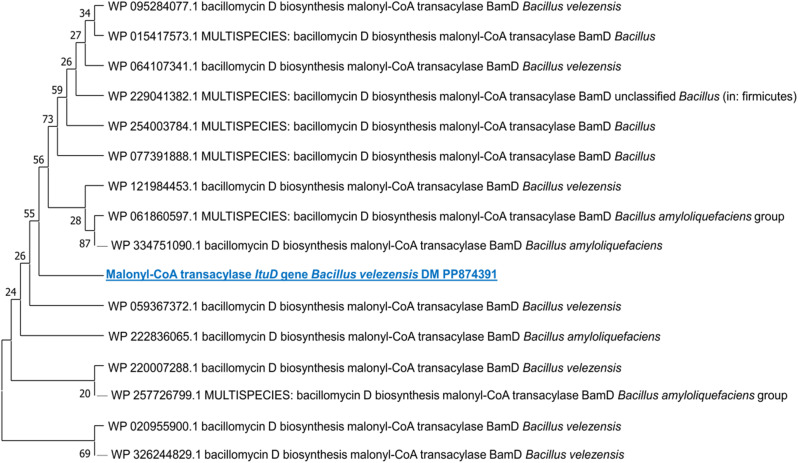
Phylogenetic analysis (1000 bootstrap replicates) of lipopeptide *ItuD* gene from *B. velezensis* DM compared with various lipopeptide *ItuD* fragments of *Bacillus velezensis*

Based on the molecular investigation, it was found that the DNA template from the rhizosphere strain, *B. velezensis* DM, successfully amplified the PKS KS domain. Furthermore, PCR amplification confirmed the presence of two lipopeptide genes for surfactin (4’-phosphopantetheinyl transferase) and iturin A (malonyl-CoA transacylase) in the same strain (Fig. 2). This confirms the biological activity of the strain. Additional PCRs were conducted using a gradient of eight annealing temperatures to verify the initial PCR results. The amplified fragments underwent sequencing to confirm the presence of the PKS KS domain and lipopeptide genes. All unique clones with the appropriate size (approximately 680 bp for the PKS KS domain, 675 bp for the *sfp* gene, and 647 bp for the *ItuD* gene) were sequenced. A BLAST analysis was conducted in GenBank (blastx), which indicated that the PKS KS fragments from *Bacillus velezensis* DM had the highest similarity to the KS fragments of *Bacillus subtilis* (SAJ35055), with a percentage identity of 99.01%. Additionally, the lipopeptide *sfp* and *ItuD* fragments from the same strain bear resemblances to the fragments of *Bacillus* sp. (WP_032726545) and *Bacillus velezensis* (WP_220007288) (99.51% and 84.93%, respectively) that are already available in GenBank as detailed in Additional file. Figures. (3, 4 and 5) illustrate phylogenetic trees created to compare the KS domains and lipopeptide *sfp* and *ItuD* genes of various microorganisms.

The sequences studied have been deposited in the NCBI GenBank database and can be accessed by the following accession numbers: PP842717 for the PKS gene, PP842718 for the *sfp* gene, and PP874391 for the *ItuD* gene.

### Structure elucidation of the isolated compound 1

**Fig. 6:**
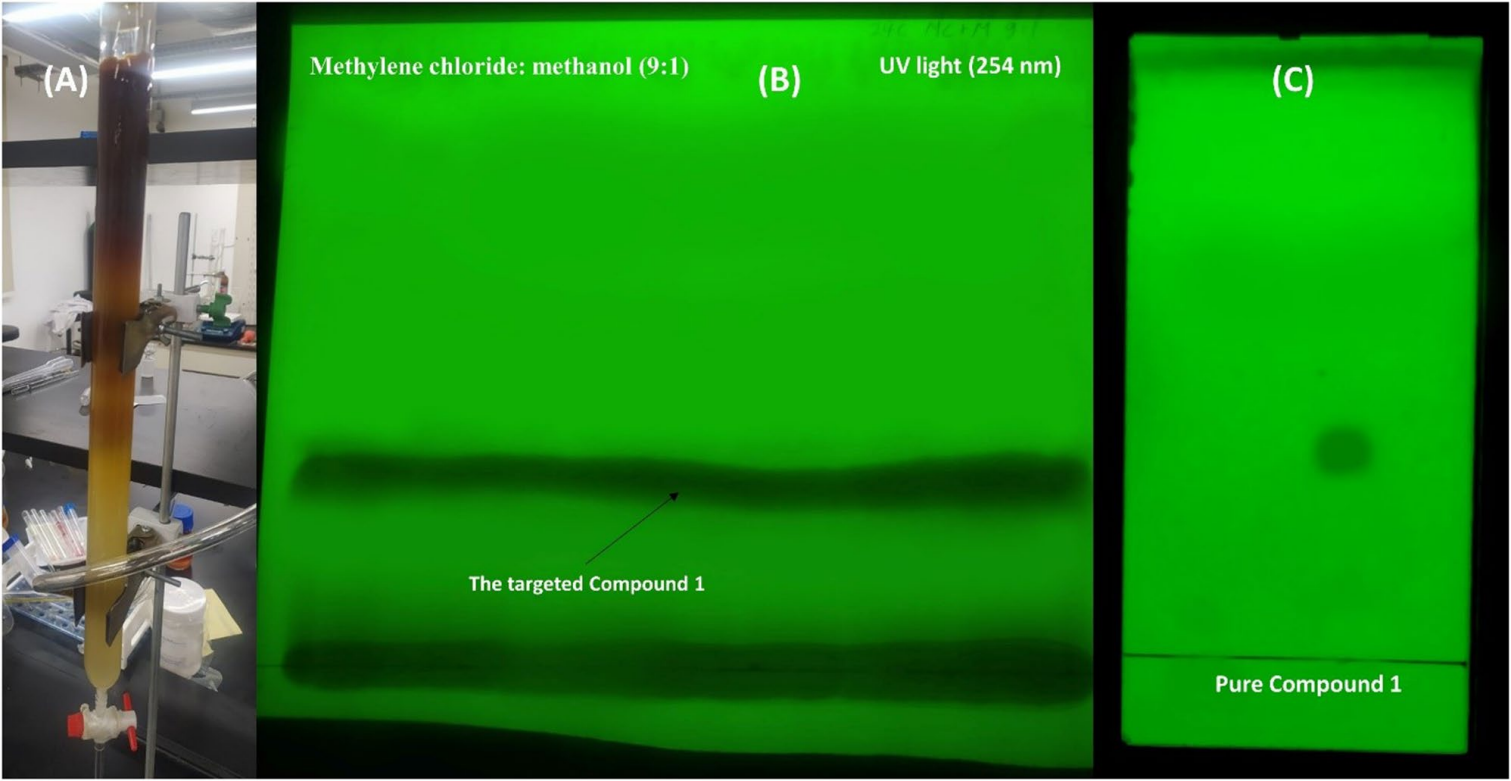
Various chromatography methods employed to isolate and purify the targeted compound **1**. [**A**] The total crude extract undergoes column chromatography, [**B**] Preparative TLC. [**C**] TLC and detection of the isolated compound **1** under UV light (254 *nm*)

One pure compound was successfully separated after subjecting the bacterial crude extract to fractionation and purification through various chromatographic techniques. This compound (9.0 mg) has R_f_ = 0.38 using a solvent system composed of methylene chloride: methanol (9:1) and visualized under short wavelength UV light (Fig. 6). When dissolved in methanol, the compound displayed UV absorbance λ_max_ at 220 nm. It gives an orange spot when spraying with Dragendorff’s reagent. ^1^H and ^13^C-NMR of compound 1 (δ in ppm, *J* in Hz) are compiled in Table 2.

**Table 2:** ^1^H and^13^CNMR of compound **1** (δ in Ppm, *J* in Hz)

Position	^1^H	^13^C
1	3.84, dd, *J* = 3.84	63.60
2	4.06, m	72.41
3	3.59, m	73.76
4	3.06, 2.94, dd, *J* = 3.08, 2.97	36.2
5	-	150.84
6	8.25	141.52
7	2.52, s	21.56
8	2.55, s	21.93

### Evaluation of the cytotoxic activity of *B. velezensis* DM extract and its pure compound 1

**Fig. 7:**
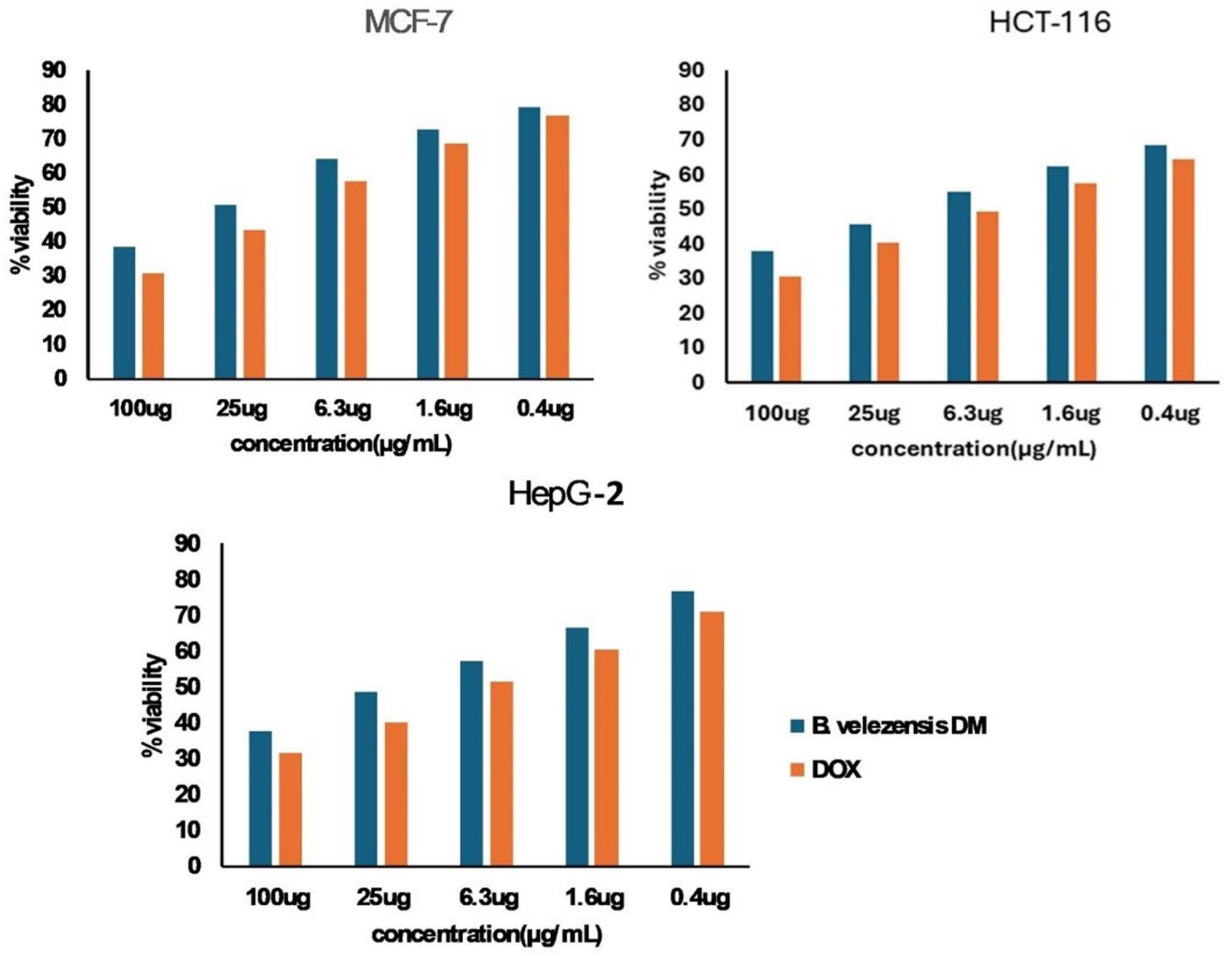
Cytotoxic effect of *B. velezensis* DM extract on different cancer cell lines (MCF-7, HCT-116, and HepG-2) shown as % viability at various concentrations (0–100 µg/mL) in comparison with the reference drug (Doxorubicin)

**Fig. 8:**
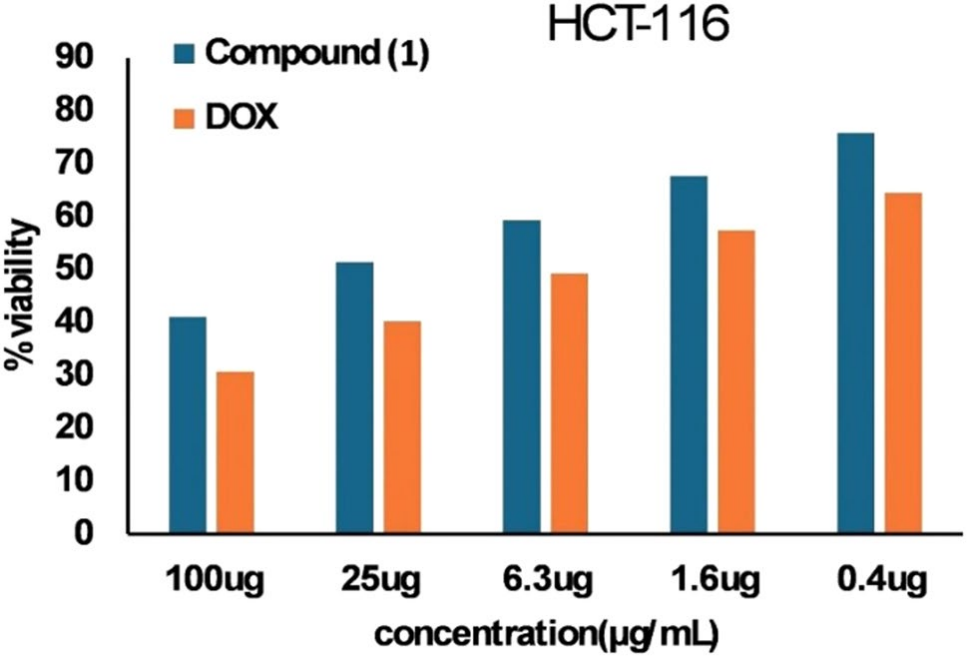
The cytotoxic effect of the isolated unique compound **1** on HCT-116 colon cancer cells in comparison with reference drug (Doxorubicin)

Figure 7 shows the effect of different concentrations (0–100 µg/mL) of *B. velezensis* DM extract on the viability of various cancer cells, including HepG-2, HCT-116, and MCF-7. As the concentration of *B. velezensis* DM extract was increased in the treated cell lines, % viable cells was decreased compared to the control cells. The lowest IC_50_ value of *B. velezensis* DM extract for cell lines was reported for HCT-116 as 12.16 ± 0.46 µg/mL, compared to DOX IC_50_ (4.82 ± 0.18 µg/mL) (Table 3). In addition, the pure compound (**1**) isolated from the same strain showed promising cytotoxicity against HCT-116 cells and good selectivity towards cancer cells (Fig. 8; Table 4).

**Table 3:** Cytotoxic activity on different cancer cell lines after treatment with *B. velezensis* DM extract

Sample	IC_50_ (µg/mL)		
	MCF-7	HCT-116	HepG-2
***B. velezensis*** **DM extract**	27.37 ± 0.94	12.16 ± 0.46	18.26 ± 1.27
**Doxorubicin (DOX)**	11.68 ± 0.4	4.82 ± 0.18	7.21 ± 0.32

**Table 4:** Cytotoxic potential of compound (**1**) on the most sensitive cancer cell line (HCT-116) and THLE-2 normal cell line

Sample	IC_50_ (µg/mL)		SI*
	HCT-116	THLE-2	
**Compound (1)**	27 ± 1.85	68.69 ± 3.34	2.54
**Doxorubicin (DOX)**	11.68 ± 0.4	17.44 ± 1.25	1.49

*Selectivityndex

### Apoptosis assay for *B. velezensis* DM extract

**Fig. 9:**
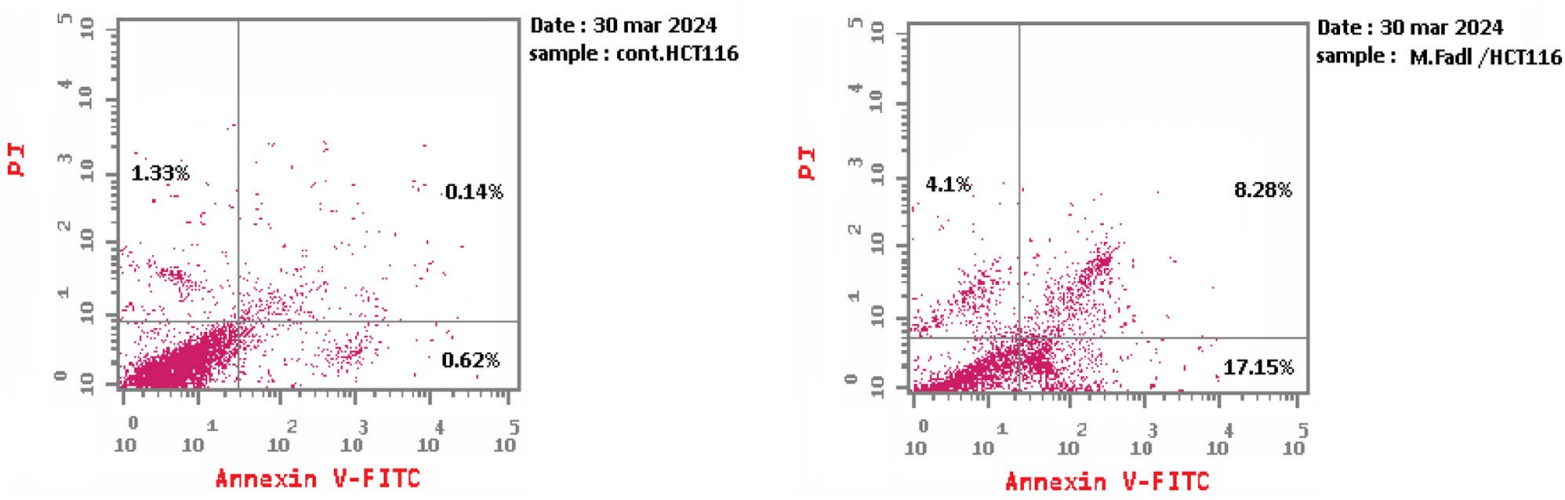
Annexin V-FITC-PI expression in HCT-116 cell line, showing the proportion of cells undergoing apoptosis in response to treatment by *B. velezensis* DM extract. Necrotic cells: upper left, late apoptosis cells: upper right, early apoptosis cells: lower right. Annexin-V binds to phosphatidylserine (PS) exposed on the surface of the early apoptotic cells, producing green fluorescence, while PI stains the DNA of late apoptotic and necrotic cells showing red fluorescence

Staining with annexin V-FITC/PI was done to determine the impact of *B. velezensis* DM extract on the induction of apoptosis in HCT-116 cancerous cells via clustering the cells into early and late apoptotic stages. The IC_50_ concentration of *B. velezensis* DM extract induced early apoptosis [annexin V (+), PI (−)], which was 17.15% compared to 0.62% in control cells, and late apoptosis [annexin V (+), PI (+)] which was 8.28% compared to 0.14% in control cells, (Fig. 9). The percentage distribution of the HCT-116 cells is summarized in Table 5.

**Table 5:** Annexin V-FITC/PI dual staining assay in HCT-116 cells after treatment with *B. velezensis* DM extract

Sample	Apoptosis %			Necrosis %
	Total	Early	Late	
***B. velezensis*** **DM extract /HCT-116**	29.53	17.15	8.28	4.1
**Control HCT-116**	2.09	0.62	0.14	1.33

### Anti-inflammatory activity of *B. velezensis* DM extract

**Fig. 10:**
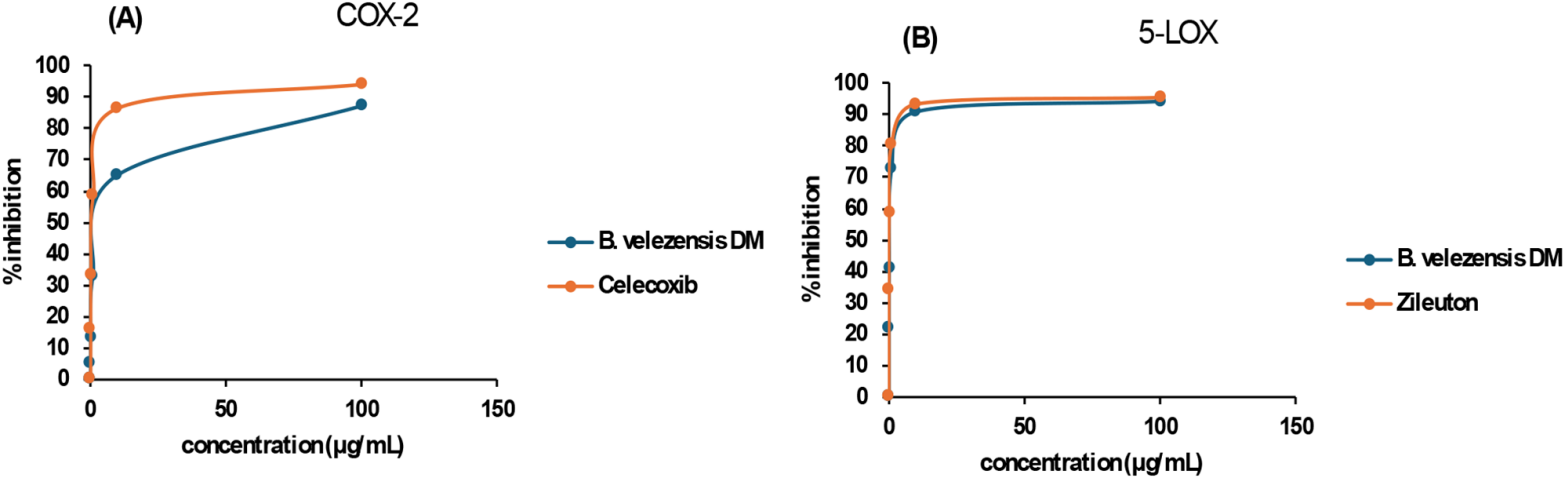
Anti-inflammatory action of *B. velezensis* DM extract illustrated via (**A**) COX-2 inhibition and (**B**) 5-LOX inhibition. Data are presented as mean ± SD

The ability of *B. velezensis* DM extract to reduce inflammation was assessed by investigating its ability to inhibit cyclooxygenase (COX-2) and lipoxygenase (5-LOX). IC_50_ values of *B. velezensis* DM extract for 5-LOX and COX-2 compared to Zileuton and Celecoxib, respectively, are shown in Table 6. Herein, *B. velezensis* DM extract displayed an anti-inflammatory action against 5-LOX and COX-2 with IC_50_ values of 1.927 ± 0.09 µg/mL and 2.672 ± 0.098 µg/mL, respectively. Results showed that as the concentration of *B. velezensis* DM extract was elevated, the degree of inhibition of 5-LOX and COX-2 activities was increased in a dose-dependent manner (Fig. 10).

**Table 6:** Anti-Inflammatory effect of *B. velezensis* DM extract via 5-LOX and COX-2 Inhibition

Sample	5-LOX (IC_50_) µg/mL	COX-2 (IC_50_) µg/mL
***B. velezensis*** **DM extract**	1.927 ± 0.09	2.672 ± 0.098
**Zileuton**	0.380 ± 0.02	--------------
**Celecoxib**	-------------	0.426 ± 0.016

### Antioxidant assessment of *B. velezensis* DM extract

**Fig. 11:**
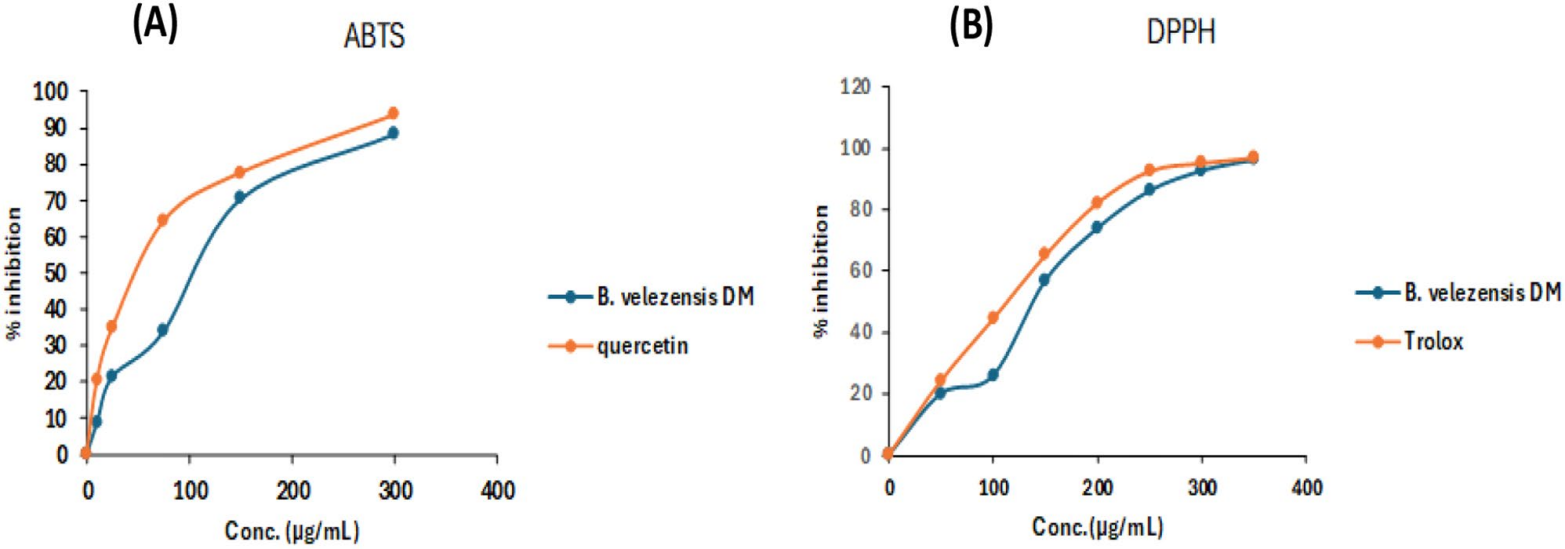
Antioxidant action of *B. velezensis* DM extract using two methods (**A**) ABTS and (**B**) DPPH. Data are presented as mean ± SD

*B. velezensis* DM extract was tested for its ability to scavenge DPPH radicals at 50–350 µg/mL concentrations. The antioxidant activity determined via scavenging DPPH radical was improved by raising *B. velezensis* DM extract concentrations. After 60 min, the highest level of RDA was 96.28% at 350 µg/mL. In addition, *B. velezensis* DM extract scavenging activity against ABTS was tested at 10–300 µg/mL. The maximum activity for ABTS scavenging activity was 88.39% at 300 µg/mL (Fig. 11). IC_50_ of *B. velezensis* DM extract against the DPPH & ABTS radicals compared to Trolox and Quercetin, respectively, are depicted in Table 7. The radical scavenging properties of the bacterial extract varied from an IC_50_ value of 147.88 ± 5.5 µg/mL against DPPH radical and IC_50_ of 76.8 ± 3.5 µg/mL against ABTS radical.

**Table 7:** Antioxidant evaluation of *B. velezensis* DM extract compared to reference drugs

Sample	DPPH (IC_50_) µg/mL	ABTS (IC_50_) µg/mL
***B. velezensis*** **DM extract**	147.88 ± 5.5	76.8 ± 3.5
**Quercetin**	--------------	42.13 ± 1.92
**Trolox**	113.31 ± 4.2	-------------

## Discussion

According to molecular characterization, it has been shown that a common ancestor gave rise to all the sequences, which eventually diverged into two distinct clusters, each composed of a particular strain of *Bacillus* sp. As a result, the investigated bacterium has been identified and designated as *B. velezensis* DM. The 16 S rDNA sequence of a specific bacterium has been deposited in the GenBank NCBI database and is now available with the accession number OR364492.

This study has revealed that many bacteria with beneficial properties for plants can be found in the roots and rhizosphere of *Datura metel* L., spanning various genera. Among them is *B. velezensis* DM, which demonstrated its ability to withstand challenging environmental conditions and was identified in this study. The current work continues the previous investigation regarding the rhizosphere of *Datura metel* L [9]. As part of the investigation into the microbiota’s diversity, certain microorganisms related to the rhizosphere of *Datura stramonium* L. were conserved [42]. Soil microorganisms are critical in facilitating various plant processes, including nutrient transformations. Among them, *Bacillus* sp. has gained significant popularity in agriculture due to its ability to form endospores that withstand high temperatures and dry conditions. In addition, *Bacillus* spp. can be processed into stable dry powders with a long shelf life [43]. Interestingly, these microorganisms are naturally found in plant root microflora and may impact the composition of plant root microbial communities [44].

The genus *Bacillus* is classified as a rhizobacterium, as it was previously discovered in the rhizosphere of different plants. This study’s taxonomic identification corresponds with recent studies identifying several *Bacillus* species. For instance, *Bacillus velezensis* strain YYC is a highly valuable rhizobacterium found in tomato plants’ rhizosphere soil [45]. Based on sequence analysis using 16 S rRNA, research findings have recently identified two distinct strains of *Bacillus velezensis*. The first strain, BS1, was isolated from rhizosphere soil in a pepper field [46]. The second strain, endophytic *Bacillus velezensis* 8 − 4, was discovered in healthy potatoes and identified through morphological characteristics and molecular analysis [47].

To explore the presence of genes responsible for secondary metabolite biosynthesis in bioactive bacteria, the PKS gene of a bacterial strain that exhibited notable pharmacological properties in our investigation was screened using degenerative oligonucleotide primers. This biosynthetic system, commonly occurring in microorganisms, yields various biologically active compounds. To confirm the presence of the PKS system in the bioactive bacterial strain, PCR primers were designed to amplify the KS domain of PKS. The screening results confirmed the presence of this system. This result aligns with the hypothesis that bioactive bacteria should possess genes specific to PKS. Hence, the strain’s major structural classes were determined to be polyketides.

Therefore, PKS genes within a particular strain may imply a robust association with the host. Previous study has revealed that certain bacteria and their host share a unique relationship that benefits both parties, aiding their survival and adaptation in their living environment. The host supplies vital nutrition, such as vitamins and nutrients, to the associated bacteria, while the bacteria excrete specific products, such as antibiotics, to enhance the host’s chemical defensive abilities [48]. To clarify the ecological importance of secondary metabolites in microbe-host interactions, the initial step involves identifying the connection between a specific bacterial strain and its host. A combination of bioactivity screening and secondary metabolite biosynthetic gene screening is essential to distinguish the biosynthetic gene in bioactive bacteria. Furthermore, it is recommended that conserved sequences from other biosynthetic pathways be integrated into PCR screening [23].

In addition, the genome of *Bacillus velezensis* DM, a strain found in the rhizosphere, underwent amplification screening for lipopeptide genes. Results have confirmed the presence of antimicrobial lipopeptide genes. This discovery is particularly significant as it indicates that *B. velezensis* DM could secrete antimicrobial lipopeptides in the rhizosphere of *Datura metel* L., which may protect the plant against a range of phytopathogens. *Bacillus* sp. produces vital antimicrobial compounds called lipopeptides, which include surfactins and iturins. The production of these lipopeptides relies on specific factors, including 4’-phosphopantetheinyl transferase and malonyl-CoA transacylase, which are encoded by the *sfp* and *ItuD* genes, respectively [22]. Previous studies have found that *Bacillus* species secrete iturin as potent antifungal compounds, which suppress the growth of filamentous fungi by disrupting sterols, phospholipids, and oleic acid in fungal membranes [21, 49, 50]. Meanwhile, surfactins have been found to exhibit potent antibacterial properties [22]. Along with these lipopeptide families, *Bacillus* species have also been reported to produce several other lipopeptides, such as surfactin-like bamylocin A from *Bacillus amyloliquefaciens*, kurstakin from *Bacillus thuringiensis*, maltacines from *Bacillus subtilis*, and polymyxins from *Bacillus polymyxa* [51–54].

**Fig. 12:**
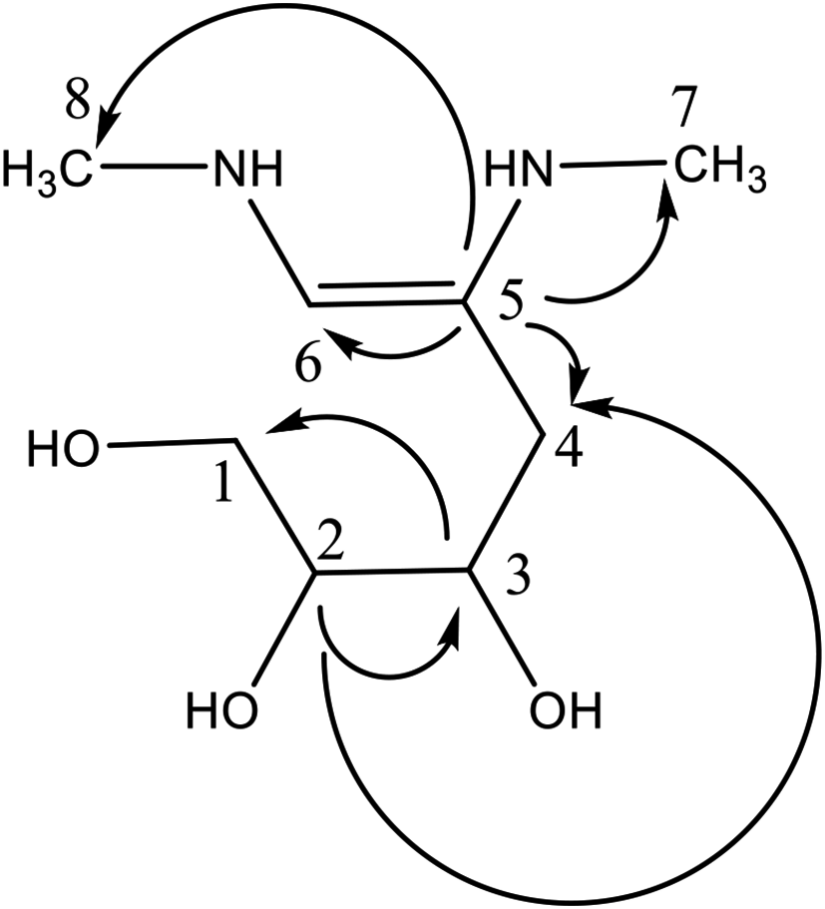
HMBC correlations of compound **1**

Compound 1 showed a molecular ion peak (ESI-MS, positive mode) at m/z 213.2957 [M^+^+Na] and m/z 235.0765 [M^+^+2Na] corresponding to the molecular formula C_8_H_18_N_2_O_3_. The carbon chemical shift found in the HMQC spectrum (Additional file) shows the presence of 2 methyl groups at δ 21.56, 21.93 respectively, one aliphatic methylene group at δ 36.2 and another one in the hydroxyl group region at δ 63.60. In addition to 2 hydroxy methine groups at δ 72.41 and 73.76 and another olefinic methine carbon at δ 141.52. The APT spectrum shows the presence of one quaternary carbon at δ 150.84. The HMBC spectrum showed many proper cross-peaks between C_3_-H_1_, C_2_-H_3_, C_2_-H_4_, C_5_-H_6_, C_5_-H_4_, C_5_-Me at H_7_, C_5_-Me at H_8_. The only double bond between C_5_ (quaternary, δ 150.84) and C_6_ (methine, δ 141.52). As a result, compound **1** could be identified as 5,6-di(methylamino)hex-5-ene-1,2,3-triol (Fig. 12). To the best of our knowledge, compound 1 is a novel natural product, as the literature lacks any information about it.

In the current work, *B. velezensis* DM extract had the potential to be an anticancer agent as it displayed cytotoxicity against the tested cancer cells, and the most potent cytotoxicity was against HCT-116 (IC_50_, 12.16 ± 0.46 µg/mL). In comparison with a recent study, the exopolysaccharide EPSF6 molecule was isolated from *Bacillus velezensis* AG6. It displayed considerable anticancer activity against HepG-2, HCT-116, and MCF-7 cell lines with IC_50_ values of 471.88, 1089, and 483.54 µg/ml, respectively [55], which is remarkably higher than that of *B. velezensis* DM extract against the same cell lines. Accordingly, the organic ethyl acetate extract derived from *Bacillus velezensis* MBTDLP1 demonstrated significant inhibitory activity against breast carcinoma (MCF-7) cell proliferation, with an IC_50_ of 0.03 mg/mL. However, it exhibited lower cytotoxicity towards normal fibroblast (3T3L) cells, with an IC_50_ of 0.14 mg/mL [14]. As a result, *B. velezensis* DM could be employed to get a unique medication against malignant human cancer cells.

Regarding 5,6-di(methylamino)hex-5-ene-1,2,3-triol (compound **1**), a novel natural compound isolated from the rhizosphere strain *Bacillus velezensis* DM, it belongs to amino alcohols. The most prevalent group of naturally occurring compounds featuring a β-amino alcohol unit are hydroxy amino acids. β-amino Alcohols are valuable intermediates for preparing several biologically active compounds besides their role in the pharmaceutical industry [56]. β-amino alcohol has strong antimalarial, antibacterial, and significant anticancer activities [57]. In the current work, compound **1** was screened for its cytotoxic activities against the most sensitive tested cell line viz. HCT-116. Compound **1** demonstrated potential as a growth inhibitory agent against the human HCT-116 cancer cells compared to Doxorubicin with higher selectivity towards cancer cells. Thus, the cytotoxicity study of the anticancer activity of compound **1** revealed that it was notably cytotoxic to cancer cells and significantly less toxic to the normal cells compared to the standard cytotoxic drug.

Annexin V-FITC/PI studies for apoptosis assessment revealed the quantitative measure of viable, apoptotic, and necrotic cells. They proved the apoptotic effect induced by *B. velezensis* DM extract in HCT-116 cells. Colon cancer cells treated with this bacterial extract displayed much less viability, showing up to a 59-fold increase in the cells at the late apoptosis stage and about a 28-fold increase in the cells at the early apoptosis stage. These results are consistent with the findings of the apoptosis assay for another study, as the organic extract of the same strain, *B. velezensis* MBTDLP1, successfully induced apoptosis in 81% of the cancer cells while maintaining nearly 60% viability in normal cells [14]. Lastly, the presented results indicate that *B. velezensis* DM has the potential to yield bioactive compounds with therapeutic and biomedical applications.

The anti-inflammatory activity of *Bacillus velezensis* is significantly linked to its ability to inhibit the key pro-inflammatory enzymes 5-lipoxygenase (5-LOX) and cyclooxygenase-2 (COX-2), which are central mediators of inflammatory pathways. According to the current findings mentioned above, it was found that *B. velezensis* DM extract has anti-inflammatory action, which agrees with the anti-inflammatory efficiency of the exopolysaccharides produced by the polluted soil bacteria [58]. In comparison with a recent study, it was found that the average IC_50_ values for exopolysaccharide EPSF6 derived from *Bacillus velezensis* AG6 on 5-LOX and COX-2 were 14.21 ± 1.20 and 16.82 ± 1.01 µg mL^− 1^, respectively [55], which are significantly higher than that of *B. velezensis* DM extract presented in this study. Furthermore, heterotroph *Bacillus velezensis* MBTDLP1, which was associated with Marine macroalga *Laurencia papillosa*, displayed prospective anti-inflammatory activity (IC_50_ 0.01 mg/mL against 5-lipoxygenase) [14]. Studies have demonstrated that *B. velezensis* produces bioactive compounds, including exopolysaccharides (EPSs), which exhibit strong dual inhibition of 5-LOX and COX-2, thereby reducing the production of inflammatory mediators such as leukotrienes and prostaglandins [59]. Another heteroacidic EPS from *B. velezensis* displayed inhibitory activity with an IC_50_ of 14.21 ± 1.20 µg/mL for 5-LOX inhibition and 16.82 ± 1.01 µg/mL for COX-2 inhibition [60]. Another study highlighted that *B. velezensis*-derived surfactin effectively downregulated COX-2 gene expression, thereby reducing the levels of prostaglandin E2 (PGE2), a key driver of inflammation [61]. Moreover, *B. velezensis* extracts have been found to suppress nitric oxide (NO) production, further contributing to its anti-inflammatory effects [62]. These findings highlight the therapeutic potential of *B. velezensis* in managing inflammatory diseases through a dual-action mechanism targeting both 5-LOX and COX-2 pathways.

Regarding the antioxidant assessment using DPPH and ABTS, *B. velezensis* DM extract showed strong RSA ability. Higher antioxidant activity was seen against ABTS in comparison to DPPH, which may be attributed to the fact that ABTS assesses the antioxidant capabilities of the extracts with both hydrophilic and hydrophobic properties. In contrast, DPPH assesses the antioxidant capacity of hydrophobic components [63]. Research findings have indicated that the exopolysaccharide EPSF6 derived from *Bacillus velezensis* AG6 exhibited a considerable decrease in IC_50_ value (100 µg/mL) against the DPPH radical. Still, it showed a significant rise in IC_50_ (500 µg/mL) against ABTS [55], compared to that of *B. velezensis* DM extract. Likewise, it was reported that exopolysaccharides from *B. velezensis* had strong antioxidant effects against DPPH and ABTS [64]. In addition, the antioxidant activity of *B. velezensis* DM extract was found to be higher than that of *Bacillus velezensis* MBTDLP1, which displayed IC_50_ values at 896 ± 0.03 µg/mL for DPPH radical scavenging and 107 ± 0.08 µg/mL for ABTS radical scavenging [14]. The anti-inflammatory and antioxidative properties of bioactive constituents might constitute potential pharmacophores against several disorders.

## Conclusion

Due to the vast reservoir of rhizosphere bacterial communities, this study reveals various findings in the therapeutic use of bioactive metabolites. A potent bacterial strain was isolated from the rhizosphere of *Datura metel* L. and successfully identified through molecular analysis. Its 16 S rRNA gene sequence has been recorded in the NCBI GenBank database under accession number OR364492, and it has been identified as *Bacillus velezensis* DM. PCR screening and sequencing analysis show that the bacterial strain possesses PKS and two lipopeptide genes. The sequences presented in this study have been deposited in GenBank with obtained accession numbers. Using diverse chromatographic methods and structure elucidation techniques, the ethyl acetate fraction derived from *B. velezensis* DM provided a new compound identified as 5,6-di(methylamino)hex-5-ene-1,2,3-triol (**1**). *B. velezensis* DM exhibited promising potential as a therapeutic agent, capable of providing antioxidant, anti-inflammatory, and anticancer efficiencies. Future studies are recommended to characterize more bioactive compounds from *B. velezensis* DM and develop prospective bioactive agents for treating life-threatening human diseases.

## Electronic supplementary material

Below is the link to the electronic supplementary material.

Supplementary Material 1

## Data Availability

The datasets generated or analyzed during the current study are available in the NCBI GenBank database with the accession numbers: OR364492, PP842717, PP842718 and PP874391.

## Abbreviations

D. metel L.: Datura metel L.
B. velezensis: Bacillus velezensis
DM: Strain code
PKS: Polyketide synthase
sfp: >4’-phosphopantetheinyl transferase
ItuD: Malonyl-CoA transacylase
PTLC: Preparative Thin Layer Chromatography
DCM: Dichloromethane
MeOH: Methanol
CDCl_3_: Deuterated chloroform
DMSO: Dimethyl sulfoxide
MTT: 3-(4,5-dimethylthiazol2-yl)-2,5-diphenyltetrazolium bromide
HepG-2: Hepatocellular cancer cell line
HCT-116: Colorectal cancer cell line
MCF-7: Breast cancer cell line
THLE-2: Normal epithelial cell line
SI: Selectivity index
IC_50_: Half maximal inhibitory concentration
EPS: Exopolysaccharide
PGE2: Prostaglandin E2
