# Supplementary material for: Pharmacological assessment of the extract and a novel compound of Bacillus velezensis DM derived from the rhizosphere of Datura metel L. with microbial molecular screening
Source: BMC Complement Med Ther. 2025 Apr 26;25:160. doi: 10.1186/s12906-025-04879-x (PMC12032720; doi:10.1186/s12906-025-04879-x)
Supplement: Supplementary file 1 — Supplementary Material 1 [file 12906_2025_4879_MOESM1_ESM.docx]

**Pharmacological assessment of the extract and a novel compound of *Bacillus velezensis* DM derived from the Rhizosphere of *Datura metel* L. with Microbial Molecular Screening**

Mohamed A. Awad^1,2^

mohamed.abo-elfadl@ejust.edu.eg

Shahenda Mahgoub^3^

shahenda.mahgoub@pharm.helwan.edu.eg

Hesham S. M. Soliman^4,6*^

[hesham.soliman@ejust.edu.eg](mailto:hesham.soliman@ejust.edu.eg)

Sherif F.Hammad^5,6^

sherif.hammad@ejust.edu.eg

^1^Biotechnology Program, Institute of Basic and Applied Science, Egypt-Japan University of Science and Technology (E-JUST), New Borg El-Arab City, 21934 Alexandria, Egypt.

^2^Botany and Microbiology Department, Faculty of Science, Sohag University, 82524 Sohag, Egypt.

^3^Department of Biochemistry and Molecular Biology, Faculty of Pharmacy, Helwan University, Ain-Helwan, Cairo 11795, Egypt

^4^Department of Pharmacognosy, Faculty of Pharmacy, Helwan University, Ain‑Helwan, Cairo 11795, Egypt.

^5^Department of Pharmaceutical Chemistry, Faculty of Pharmacy, Helwan University, Ain‑Helwan, Cairo 11795, Egypt.

^6^PharmD Program, Egypt-Japan University of Science and Technology (E-JUST), New Borg El-Arab City, 21934 Alexandria, Egypt.

* Corresponding author: [hesham.soliman@ejust.edu.eg](mailto:hesham.soliman@ejust.edu.eg)

**PCR detection of genes related to bioactive metabolites biosynthesis in *Bacillus velezensis* DM**


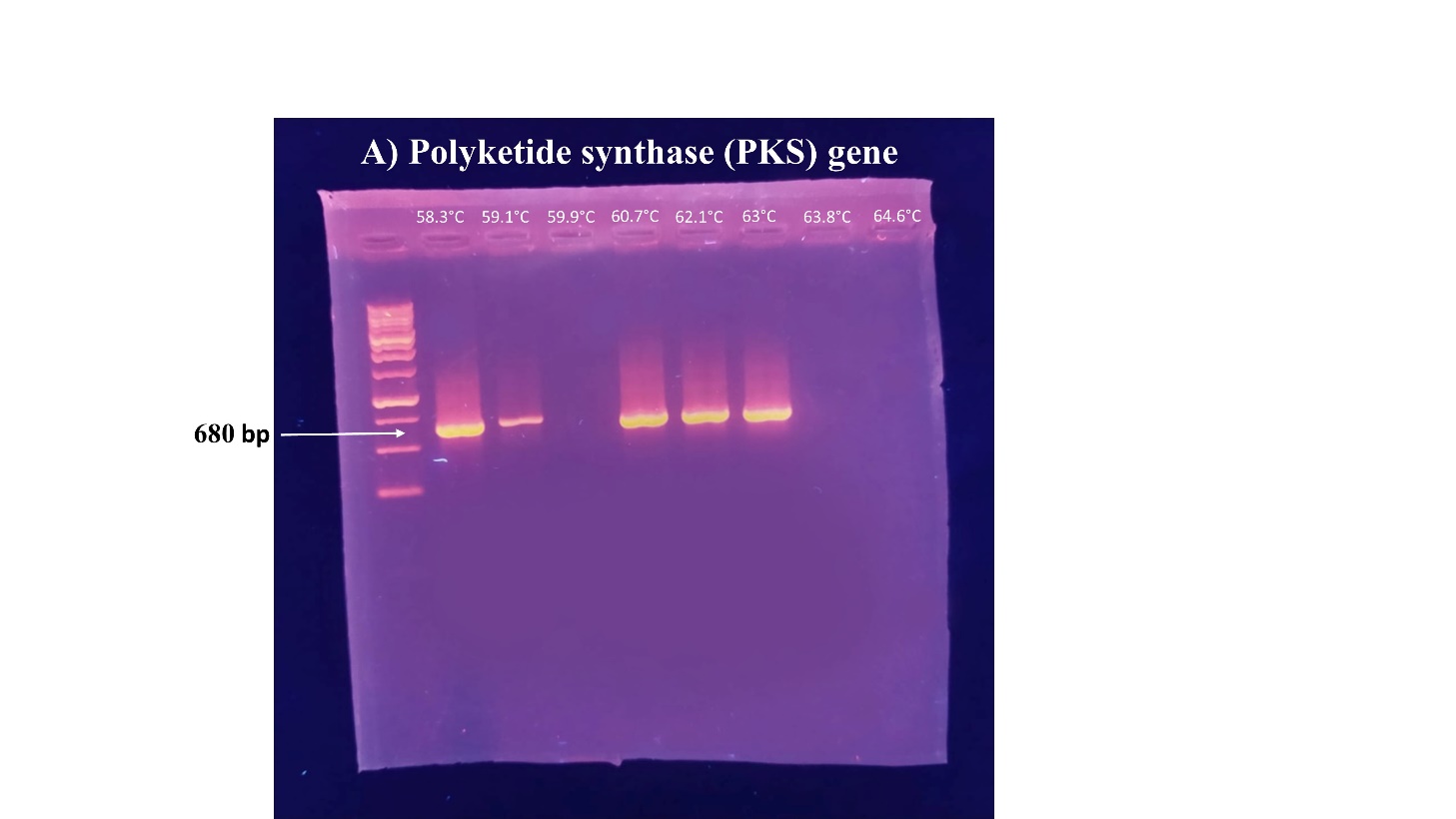


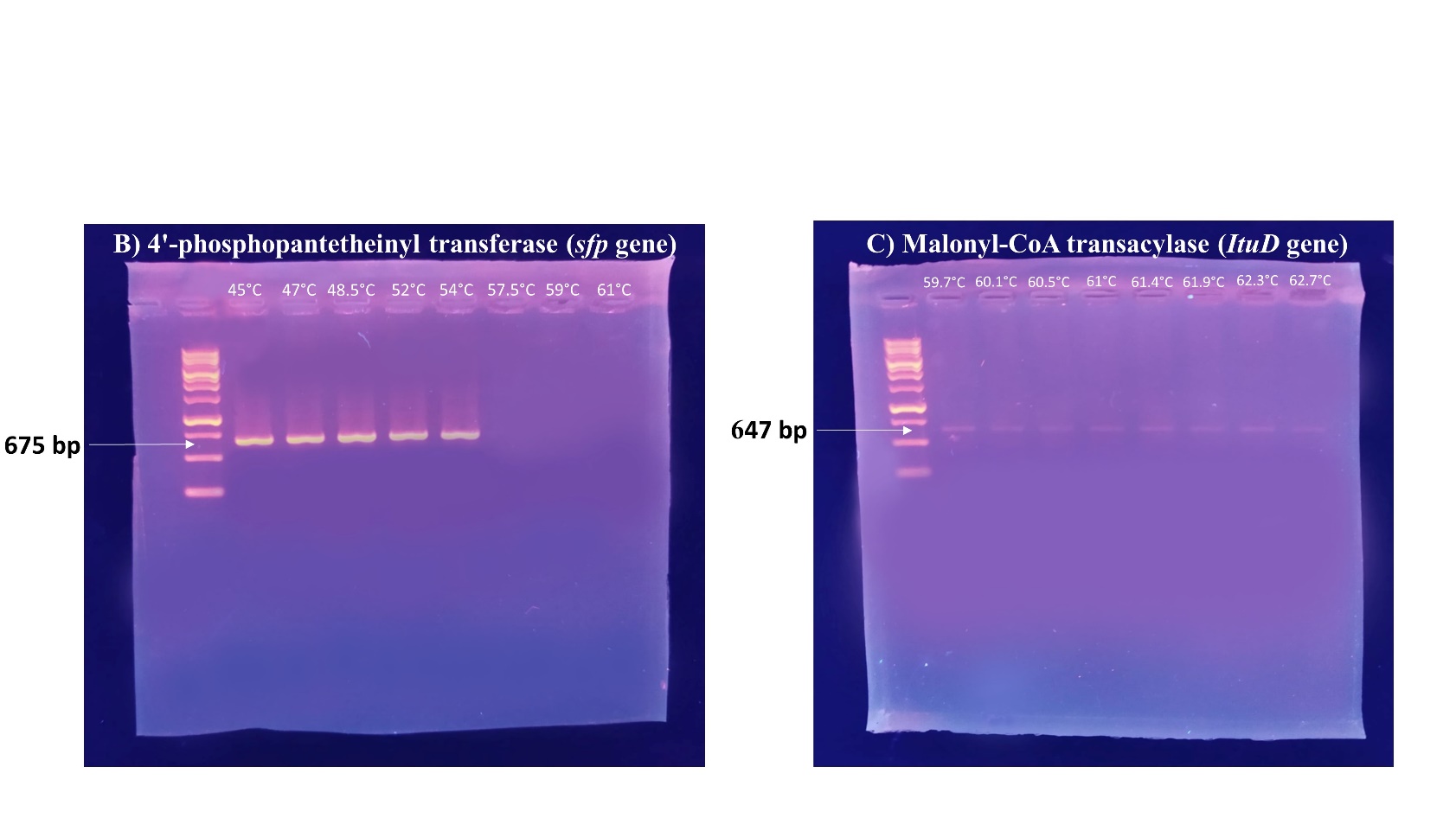


**Fig. S1** PCR amplification detects the presence of some genes related to the biosynthesis of bioactive metabolites in the rhizosphere strain, *Bacillus velezensis* DM, on a 1% agarose gel electrophoresis using a gradient of eight annealing temperatures to verify the initial PCR results as follows: Polyketide synthase which encodes KS domain (**A**) and lipopeptide genes for surfactin and iturin A, including 4'-phosphopantetheinyl transferase (**B**) and malonyl-CoA transacylase (**C**), respectively.

**BLAST Results**

**Table 1** NCBI BLAST results showing identity between 16S rRNA gene sequence from *B. velezensis* DM and other most related sequences available on GenBank database.


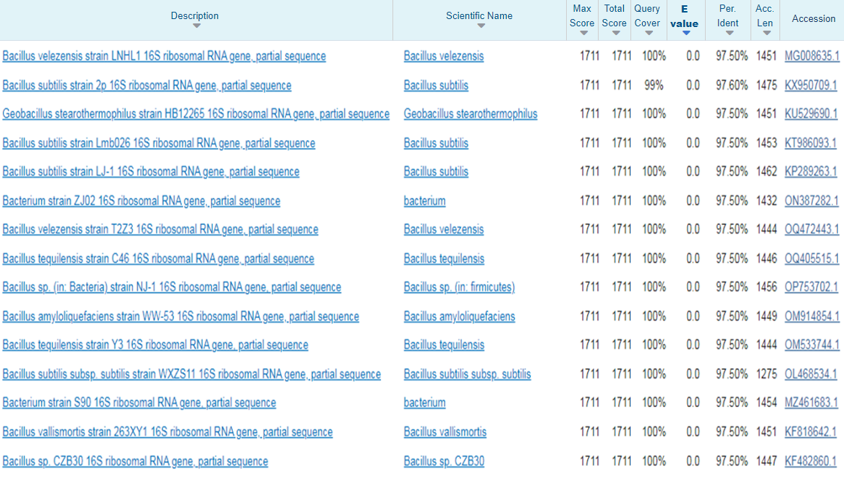


**Table 2** NCBI BLAST results showing identity between PKS gene sequence from *B. velezensis* DM and other most related sequences available on GenBank database.

**
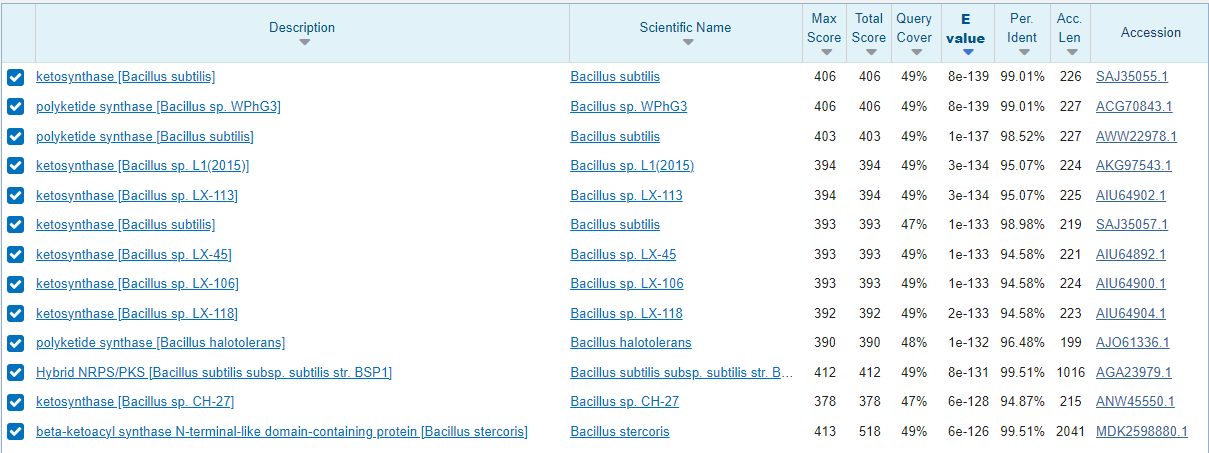
**

**Table 3** NCBI BLAST results showing identity between lipopeptide *sfp* gene sequence from *B. velezensis* DM and other most related sequences available on GenBank database.


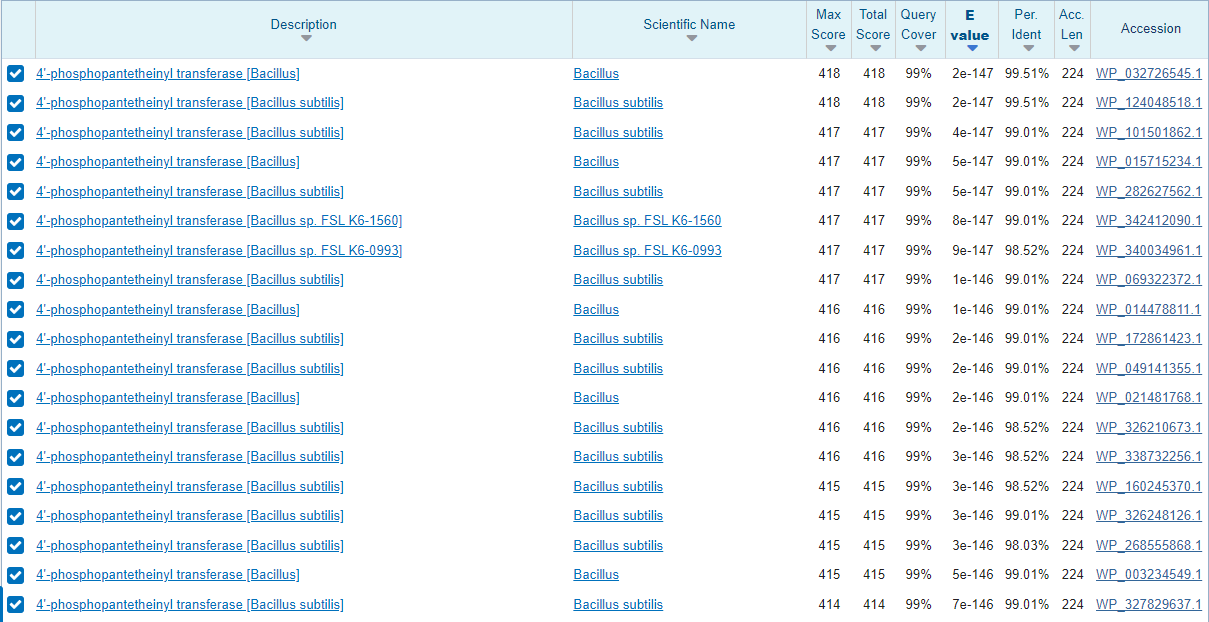


**Table 4** NCBI BLAST results showing identity between lipopeptide *ItuD* gene sequence from *B. velezensis* DM and other most related sequences available on GenBank database.


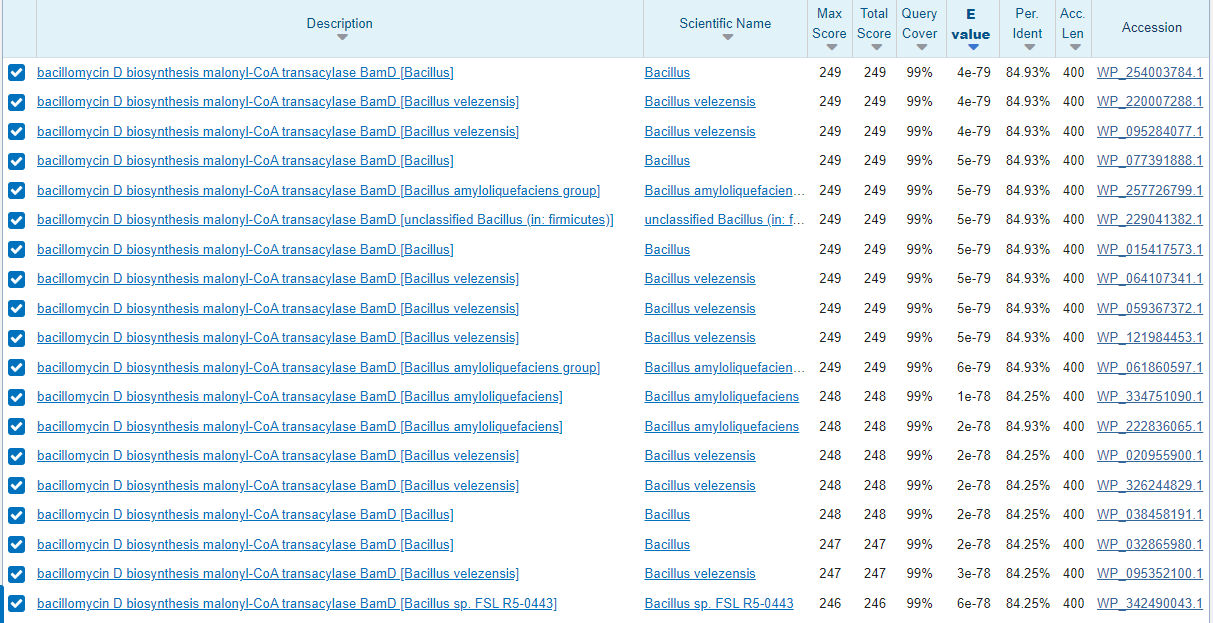


**Structure Elucidation of Compound 1**

**
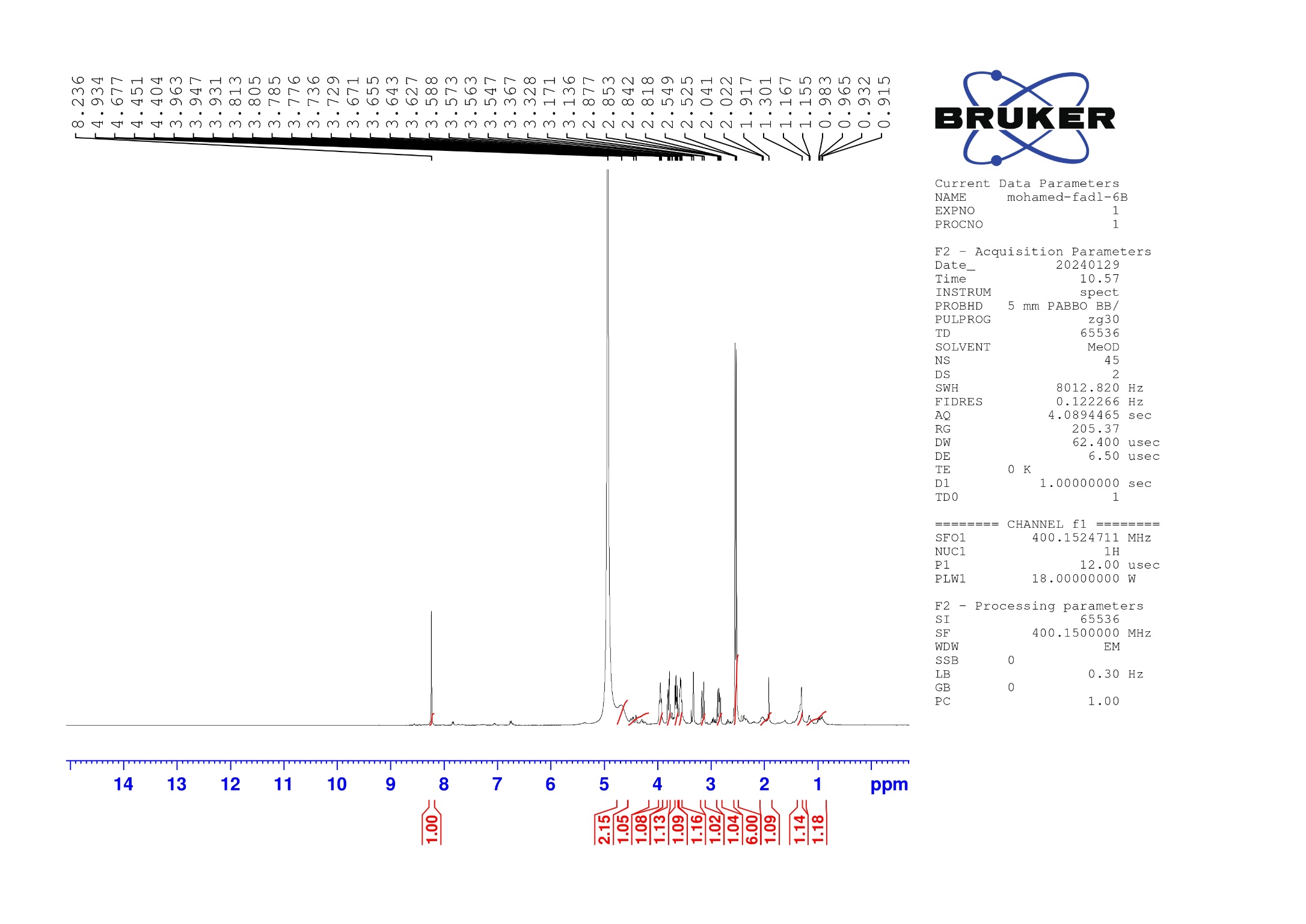
**

**Fig. S2.** ^1^H NMR (400 MHz, MeOD) spectrum of compound **1**

**
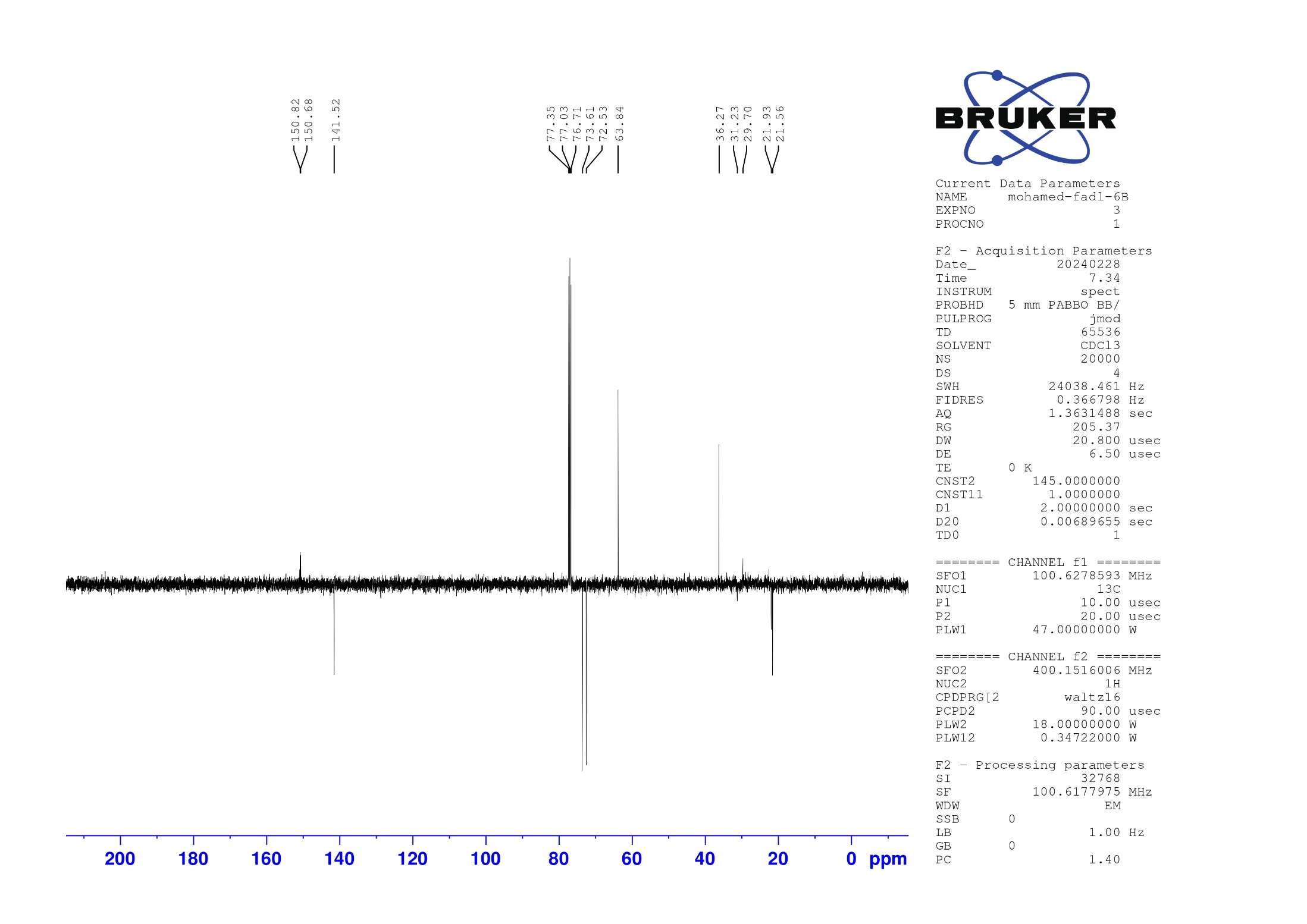
**

**Fig. S3.** APT ^13^C NMR (100.63 MHz, CDCl3), spectrum of compound **1**

**
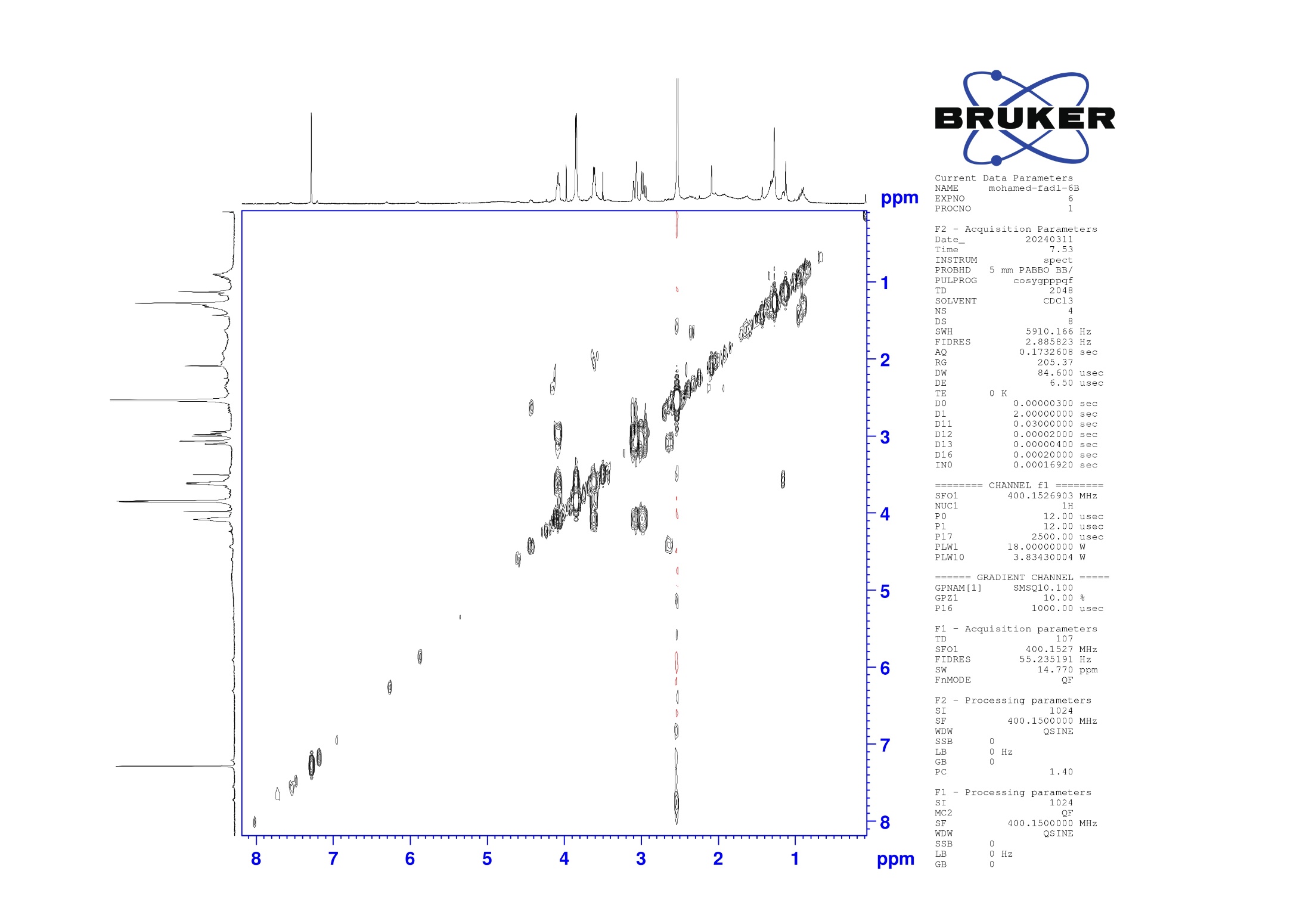
**

**Fig. S4.** H-H COSY spectrum of compound **1**

**
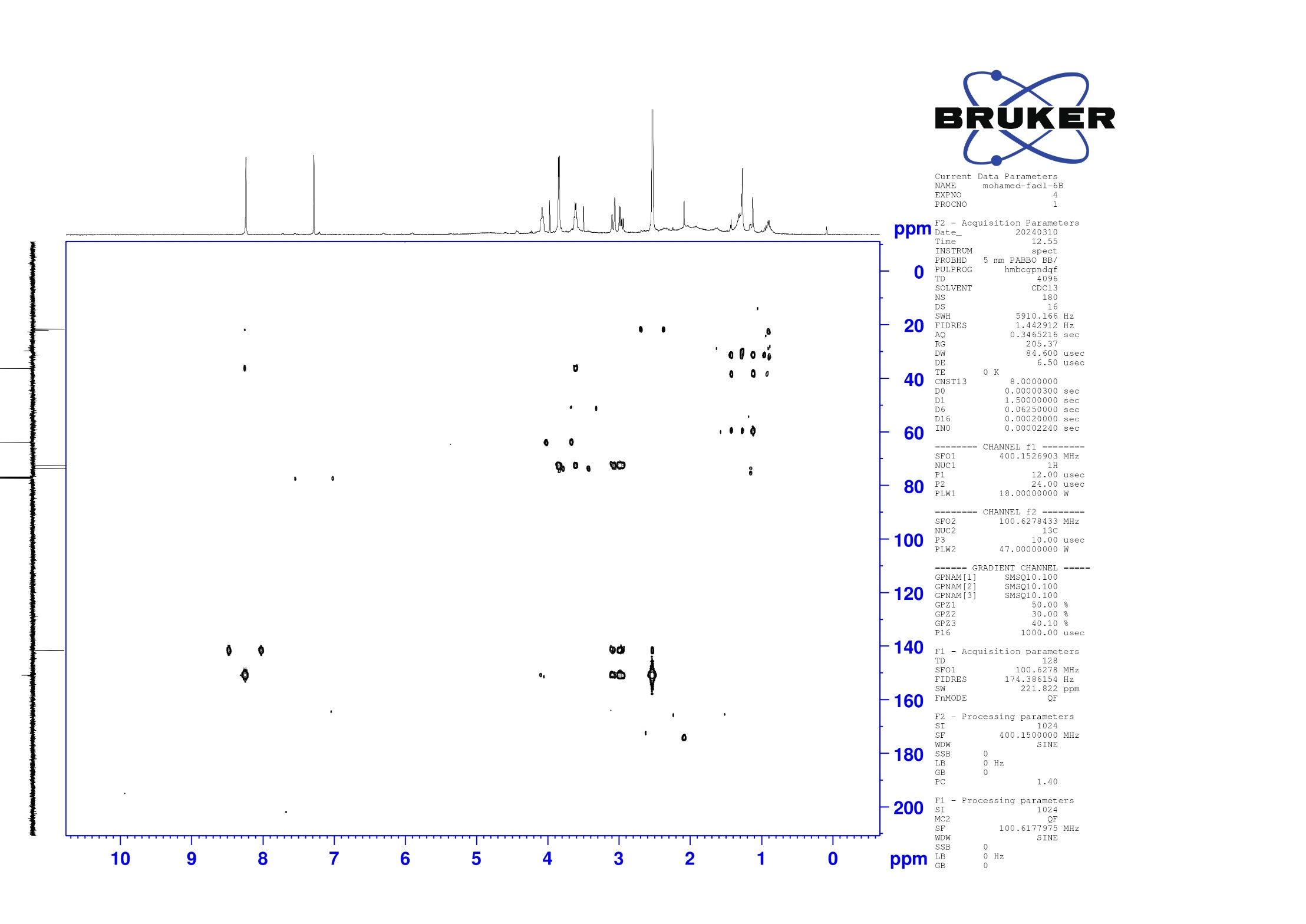
**

**Fig. S5.** HMBC spectrum of compound **1**

**
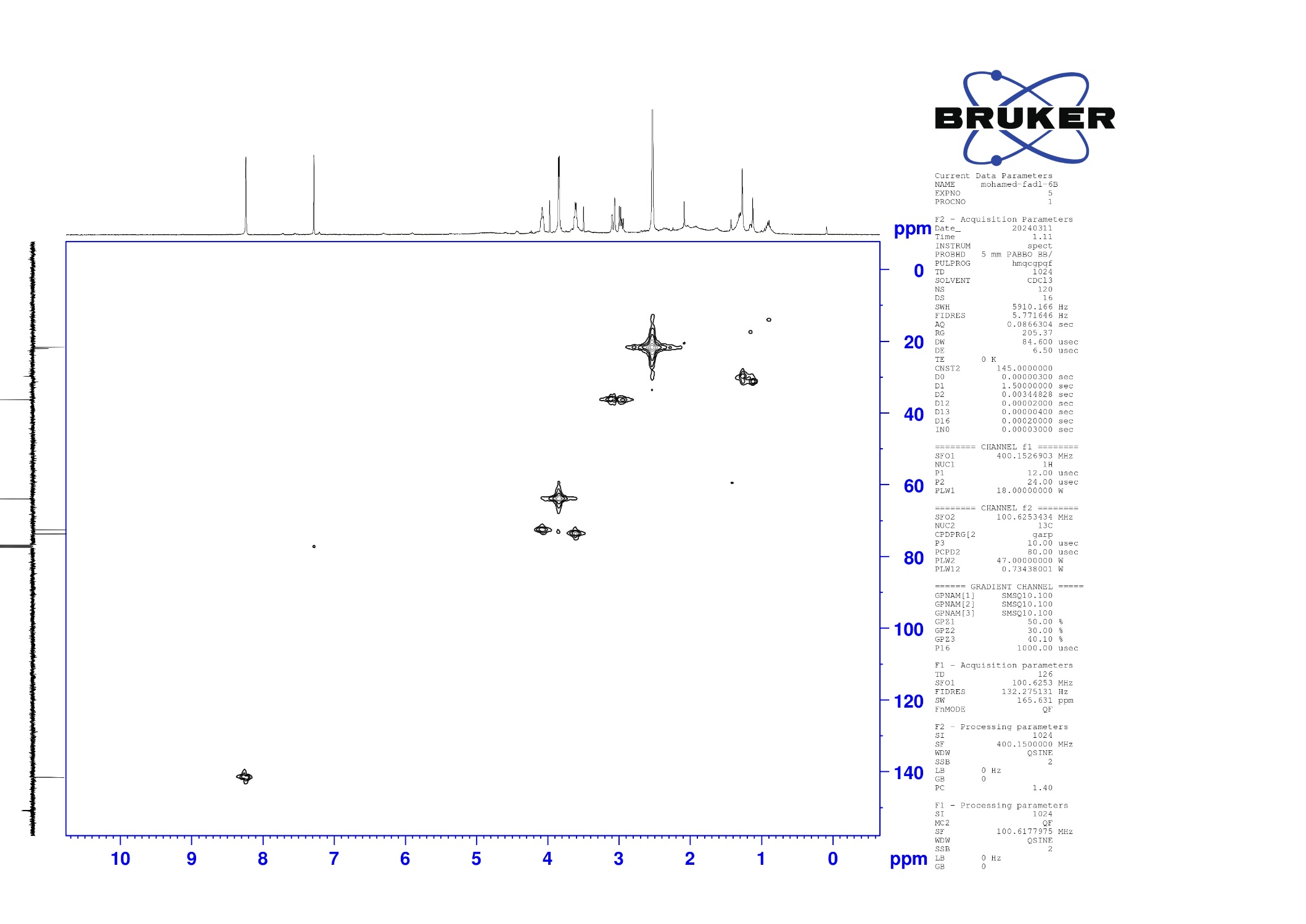
**

**Fig. S6.** HMQC spectrum of compound **1**

**
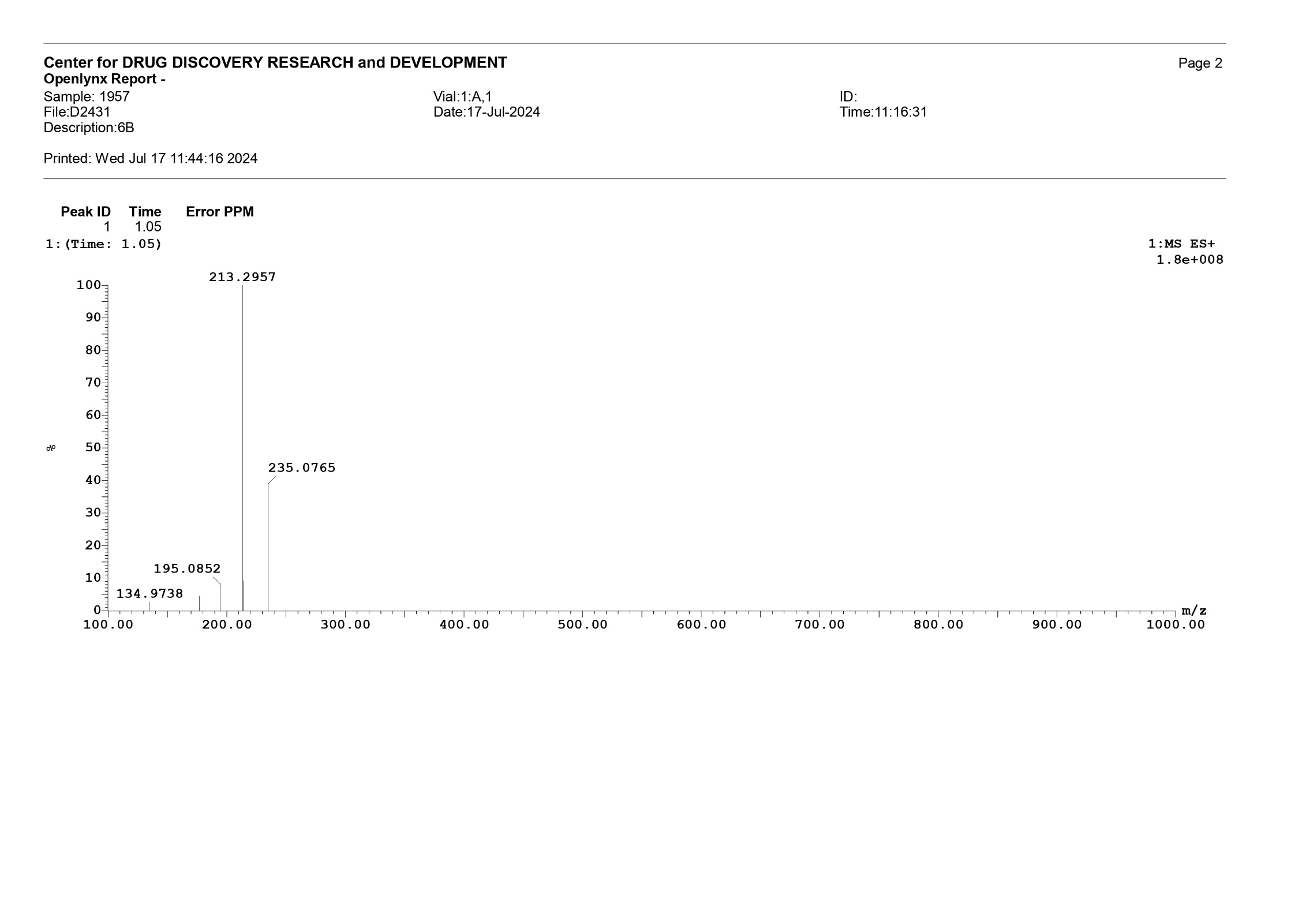
**

**Fig. S7.** (+) ESI-MS spectrum of compound **1**
